# Educators’ perceptions of organisational readiness for implementation of a pre-adolescent transdisciplinary school health intervention for inter-generational outcomes

**DOI:** 10.1371/journal.pone.0227519

**Published:** 2020-01-08

**Authors:** Keshni Arthur, Nicola Christofides, Gill Nelson

**Affiliations:** 1 School of Public Health, Faculty of Health Sciences, University of the Witwatersrand, Johannesburg, South Africa; 2 UCL Institute for Global Health, University College London, London, United Kingdom; University Lyon 1 Faculty of Dental Medicine, FRANCE

## Abstract

Organisational readiness is an implementation pre-requisite to gain its members' appropriate and committed use of the intervention. Implementation climate and organisational readiness for implementing change were evaluated conjointly to assess organisational readiness for an obesity and HIV health intervention that imparts health information directly to Grade 6 learners, and indirectly to their parents/caregivers in their home environment. The study objectives were to assess the level of organisational readiness at schools and to identify organisational factors (facilitators, barriers and contextual factors). A mixed-methods approach collected data from five public schools in Gauteng, South Africa. Forty-six educators and school management answered a self-administered questionnaire and contributed to a focus group discussion at each school. Mean scores with standard deviations, or median scores with interquartile ranges, were calculated to determine levels of organisational readiness. Qualitative data were transcribed and analysed thematically. The overall implementation climate and organisational readiness for implementing change median scores were acceptable, at 3.6 (IQR 3.2–4.1) and 4.3 (IQR 3.8–4.9), respectively. Results indicated that educators collectively valued the change highly enough to commit to its implementation, and that the motivation for the intervention, associated goals and objectives, the realisation for change, and the benefits thereof were well-comprehended by educators. Thirteen barriers and 13 facilitators were identified. The perceived degree of fit between the significance and values attached to the intervention by educators, and how these would be received by the target group (parents and learners) was also beneficial. Key barriers and facilitators indicated that the intervention needed to be a fit with existing workflows and educational systems. Contextual factors such as intervention appropriateness and acceptability as well as sensitivity to HIV were identified. These findings suggested proactive improvements to further improve the intervention and its implementation strategy.

## Introduction

Organisational readiness (OR) is an implementation strategy that addresses barriers to the introduction of new programmes by providing tools that promote the adoption and offer potential solutions to improve implementation efforts [1]. Organisational readiness of schools refers to the extent to which the organisation is both willing and able to implement an innovation [2] and is a necessary precursor to its successful implementation. Implementation of any intervention requires refinement to address emerging problems, and yield sustained efforts [3]. Interventions to prevent disease and promote health are being developed and refined as evidence grows on how best to do this.

Globally, the double burden of communicable diseases (CD) and non-communicable diseases (NCD) affects the health of populations, particularly in low- and middle-income countries. South Africa has been tackling the human immunodeficiency virus (HIV) epidemic for decades [4]; NCDs, such as diabetes and cardiovascular disease, have garnered attention more recently [5,6]. Obesity is a key contributor to the rise in NCDs and, together with HIV, is a cause of early mortality in South Africa [4]. Targeting interventions at school, learners could potentially carry knowledge learned into adulthood [7], and influence parents’ or caregivers’ health behaviours [7].

To address the double burden of disease in South Africa, *The CIrCLE (Child Influencing paRent Communication for Life Education) of Life Initiative*, was developed to address both obesity and HIV through a school health education programme that aims to increase knowledge and enhance skills among learners and their parents/caregivers. The school-based intervention is delivered through trained educators via a learning curriculum that directly targets Grade 6 pre-adolescent learners who, in turn, indirectly communicate knowledge learned to their parents. A comprehensive description of the intervention is reported elsewhere (S1 File).

*The CIrCLE of Life Initiative* required active institutional support for its implementation. Implementation research can identify barriers to, and enablers of, effective global health programming, and leverage that knowledge to develop evidence-based innovations in effective delivery approaches [8]. Implementation efforts that are informed by, and tailored to, potential barriers and facilitators, are more effective and sustainable [9]. The key factors underlying the successful implementation of a health intervention, such as *The CIrCLE of Life Initiative*, include maximising school engagement and identifying features that influence behaviour change for improved health outcomes, which need to be understood to ensure that the programme has the potential to reach both learners and parents and for the health benefits to be realised.

The implementation of an intervention requires OR [1] as well as the targeted organisational members' commitment to use the intervention [10]. Organisational readiness could be influenced by the characteristics of the educators and/or management, their social norms, and their preparedness for the intervention. When OR is high, educators are more likely to initiate change, exert greater effort, exhibit greater persistence, and display more cooperative behaviour, resulting in effective implementation of the proposed change [11]. Conversely, when OR is low, educators would be less likely to view the change as desirable, and resist planning for, and participating in, the process [11].

Implementation effectiveness is influenced by measures of the organisational context, such as OR for change, and implementation climate [12]. Implementation effectiveness is dependent on the consistency and quality of targeted organisational members' use of an innovation as a function of the fit of the innovation to targeted users' values, and the strength of an organisation's climate for the innovation [10].

Implementation climate is defined as employees’ shared perceptions of the extent to which their use of a specific innovation is rewarded, supported and expected within an organisation [10]. It is shaped by an organisation’s policies and practices. Implementers develop a collective sense of the organisation’s priorities and sanctioned means for achieving those priorities through interactions with each other and with implementation strategies [11]. Implementation climate in schools can be strengthened by providing educators with access to training, technical assistance; engaging them in decision making; offering incentives and feedback on intervention use; and increasing intervention facilitators and removing barriers [11].

Organisational readiness for change is a shared psychological state in which members feel committed to implementing a change (change commitment) and confident in their collective capability to do so (change efficacy) [2]. Both change commitment and change efficacy are influenced by how favourably educators assess implementation capability of either staff or the organisation, i.e. the task demands, resource availability, and the key features of the environment surrounding the intervention as barriers to, or facilitators for, change. Wiener (2009) states that organisational readiness for change at schools could vary as a function of how much educators value the change (change valance) and how they appraise implementation capability [2]. Researchers have tested both the OR for implementing change [13,14] and the implementation climate [15,16] constructs, but have usually only utilised one of the constructs as an outcome measure [11,15–17]. To the author’s knowledge, this paper will be the first to report the use of both constructs in one study.

Organisational readiness has been assessed in various settings [15,17,18], including nursing homes [19], workplaces [11], welfare institutions [12] and schools [16,20–23]. Studies that have conducted OR at schools have focused on capacity building [21,22], organisational implementation context [16] and school climate [23]. Factors associated with the implementation of programmes and reforms at schools include time [24,25] and resource availability [24,25]. Other implementation issues are unique to specific studies and include supportive school climate [24], the availability of staff [25] and necessary teacher skills [25]. This demonstrates that the unique features of programmes implemented in different school contexts may have common barriers and facilitators, but are also likely to have implementation issues specific to different interventions or schools.

The objectives of the present study were to assess the level of OR in schools and to identify organisational facilitators, barriers and contextual factors that might suggest proactive improvements before implementing the intervention: *The CIrCLE of Life Initiative*.

## Methods

### Study design

A mixed-methods approach [26] was employed; quantitative and qualitative data were collected concurrently and integrated during data analysis.

### Study setting and participants

South African government-run schools are categorised into five groups or quintiles [27], based on the relative wealth of their surrounding communities. Schools in the most impoverished communities are classified as quintile 1 and those serving the wealthiest communities, as quintile 5. Quintiles 1, 2 and 3 schools do not charge fees. Quintile 1 schools receive the highest subsidy from the government, and quintile 5 schools, the lowest [27]. Rural schools in the country have many more shortcomings than urban schools, due to poverty and under-development [28]. Five primary schools in Gauteng province, South Africa, were purposely selected to represent each of the quintiles. Three were in rural areas and classified as quintile 1 or 2 schools; two were urban-based quintile 3 and 4 schools.

Participants were selected through a purposive sampling method. Invited participants included all school management (headmasters, deputy headmasters and heads of department) and all Grade 6 educators, as the intervention was targeted at Grade 6 learners.

### Procedures

An information session was held with the school management and Grade 6 educators to inform them about the intervention and to discuss implementation strategies. Data were collected during the session. Forty-six participants answered a self-administered questionnaire that explored OR at their schools. A focus group discussion (FGD) comprising four to 15 participants, followed at each school lasting approximately 15–20 minutes. The interview guide had been pilot-tested with a separate group of educators and relevant changes made to the guide. FGDs were conducted privately at the school with only the participants and the researcher. All FGDs were audio-recorded and transcribed verbatim. The transcripts were returned to two participants per school for comment and/or correction. Observations of the general functioning of the school were documented using field notes.

S1 Table presents a Standards for Reporting Implementation Studies (StaRI) checklist with details regarding the implementation strategy that was used during the study [29].

The Human Research Ethics Committee of the University of the Witwatersrand approved the study (clearance certificate no. M180220).

### Measures

A 55-item questionnaire (S2 File) was designed to measure the implementation climate and OR for implementing change, and school-related characteristics such as the school quintile and socio-demographic characteristics of the participants.

The Implementation Climate Scale (ICS) [15] is a validated tool used to assess the degree of strategic organisational climate supportive of evidence-based practice (EBP) implementation. Measurement dimensions of team function in a school environment were adapted to represent the following scales: focus of the programme; openness of teaching staff; and recognition, educational support, and rewards for implementation of the programme. One scale (the selection for EBP) was excluded as it was irrelevant to this context.

The subscales: focus of the programme, openness of teaching staff, and recognition for implementation of the programme, used a 5-point Likert scale (1 = not at all, to 5 = very great extent). Educational support for implementation of the programme was scored on a five-point scale with anchors of responses graded across indicators of (1 = not at all, to 5 = extremely confident). Educator perception of impact of the programme was indicated by marking one or more tick boxes. In a previous study, a validity testing model for the ICS (the subscale rewards for EBP), had weaker correlations with the other subscales and exhibited some inconsistency in the aggregation statistic [12]. This subscale was therefore adapted to be more relevant to this context. Rewards for implementers in this intervention were not financial, and educators were instead asked to select all possible impacts of the programme.

The validated Organisational Readiness for Implementing Change (ORIC) questionnaire [14] defined in psychological terms, contained 12 items that measured educators’ change commitment and change efficacy to implement the intervention; a 5-point Likert scale (1 = strongly disagree, to 5 = strongly agree) was used. To facilitate analysis, subscales were labelled according to the scale that they addressed. The questions in the tool were adapted to the school context.

Cronbach’s alpha was calculated to assess the internal consistency of the subscales and composite scale. The Cronbach’s alpha was 0.81 for the overall ICS scale and 0.93 for the overall ORIC scale indicating good internal consistencies.

The questionnaire included open-ended questions about perceived acceptability and feasibility (e.g. “Do you foresee any problems in the implementation of this programme?”), perceived utility and sustainability (e.g. “What do you think will be critical to the success of the programme?” and “What do you think could limit the success of the programme?”), and uptake and penetration (e.g. “How would you recommend that the researcher get the staff involved and motivated to successfully carry out the implementation?” and “What further consultation or communication would you like to have from the researcher?”).

### Data analysis

Data from the questionnaires were captured using REDCap and exported into Stata 14 for statistical analysis. Educator perceptions of OR were described using proportions, mean scores (for normally distributed scales) or median scores (for non-normally distributed subscales, based on the Shapiro-Wilk test). The Kruskal-Wallis rank test was used to test differences between school, quintile, and fee-paying classifications.

All focus group discussion data were combined with the responses to the open-ended questions in the questionnaires and imported into Microsoft Excel for content analysis [30], where all data were systematically transformed into an organised and concise synthesis of key results. Following thematic analysis reported by Braun and Clarke [31], the data were reviewed for familiarity to generate initial coding. KA coded the data. The codes were grouped in themes and subthemes, after which a core category emerged. Data saturation was reached after the third FGD, with the final two FGDs being coded to confirm this. The themes were subsequently revised to ensure that they retained the core meaning evident in the initial data. The trustworthiness of the findings, i.e. the coding strategies and the framework generated by KA, was independently checked by NC. Interpretations were discussed and challenged appropriately to achieve final consensus. No bias issues were identified.

## Results

Forty-six of the 51 eligible educators (90.2%) consented to participate; their demographic characteristics are summarised in Table 1. The mean age of the educators was 44.8 years; average teaching tenure was 16.5 years. Most were female (71.7%). All had post-high school qualifications: 28.3% had teaching diplomas, and 71.7% had undergraduate or postgraduate degrees. Management positions were occupied by 41.3% of the educators.

**Table 1:** Demographic characteristics of participants and schools.

Characteristics		n	%
Sex			
	Female	33	71.7
	Male	13	28.3
Position			
	Educator	27	58.8
	Manager	19	41.3
Education			
	Diploma	13	28.3
	Undergraduate degree	18	39.1
	Postgraduate degree	15	32.6
Location of school			
	Rural	24	52.2
	Urban	22	47.8
School classification (Quintile)			
	1	17	37.0
	2	7	15.2
	3	15	32.6
	4	7	15.2

On average, there were nine participants per school (range 7–15), representing four quintiles: 37.0% from quintile 1, 15.2% from quintile 2, 32.6% from quintile 3, and 15.2% from quintile 4. Both rural and urban schools were represented.

The thematic analysis identified 13 barriers, presented in Table 2 and 13 facilitators, presented in Table 3, which were further classified into six categories: challenges/facilitators of the educator, learner, parent, system, implementation climate and ORIC.

**Table 2:** Themes identified under educator, learner, parent, system, implementation climate and organisational readiness for change challenges and recommendations of educators with illustrative quotations from participants.

Challenges as perceived by educators	Educator recommendations to mitigate challenges
**Educator Challenges**	
**Increased educator workload**	
*“Teachers already have too much workload and responsibilities and I think these factors will limit the success of the program”* (Educator, Q1)	*“Tell them more about how the program will benefit the staff and learners without adding any unnecessary workload to educators*.*”* (Educator, Q2)
**Learner Challenges**	
**Non-participation of learner**	
*“The response of the learners to programme as they play a critical role*. *Hope they understand their role*, *they do activities given to them*.*”* (Educator in management, Q1)	
**Intervention needs to be culturally sensitive**	
*“Culture is a big problem (Kids not allowed to question elders about personal matters)”* (Educator in management, Q1) *“At the school we have learners and teachers from different cultures*.*”* (Educator in management, Q1)	*“Sensitivity when dealing with learners because some come from families that are directly affected by some of the diseases mentioned in this questionnaire*.*”* (Educator in management, Q3)
**Parent Challenges**	
**Non-participation of parent**	
*“The only challenge that I see cooperation from parents*, *in terms of information getting to them*. *We communicate with parents through the learners*, *who do not get information to parents on time*.*”* (Educator in management, Q1) *“How do you ensure that parents are actively engaged into the programme apart from their kids*? *How do you ensure that this programme is beneficial to the community at large*?*”* (Educator, Q4)	*“Engaging parents of the learners*. *Making all stakeholders aware about the program*.*”* (Educator, Q1)
**Intervention not appropriate or acceptable**	
*“Some parents might not be free to communicate certain aspects of life with their kids at this age*.*”* (Educator in management, Q2)	
**Lack of knowledge of parents**	
*“Lack of knowledge*. *Our community they know little about this illnesses*. *Most of them are illiterate*.*”* (Educator in management, Q1)	*“Maybe there are more different ways of involving the learners as most of them are always absent or won’t be interested in the program and their parents as most of them are ignorant*.*”* (Educator, Q1)
**System challenges**	
**Inappropriate scheduling of implementation**	
*“Implement this January then pre-planning can assure the CIRCLE becomes part of their resources and daily planning*.*”* (Educator in management, Q4)	*“She needs to use life skills periods not after hours”* (Educator in management, Q3) *“The time to implement it (But I suggest that can be part of LO and Life Skills lessons”* (Educator, Q1)
**Scarcity of resources**	
*“As a school we do not have enough resources*.*”* (Educator, Q1) *“Resource may impede the programme to an extent*.*”* (Educator in management, Q1)	*“I don’t foresee any problems as long as they done in our followed*, *or if the resources are given to the educators if they are not available*.*”* (Educator in management, Q1)
**Sustainability of intervention**	
*“Is the government going to use the results to better prepare all stakeholders*? *Will the research find its way into our curricula*?*”* (Educator in management, Q1)	*“If only the results could be incorporated into departmental policies implementation would be smoother and effective*. *In terms of resources*, *this is always a challenge*.*”*(Educator in management, Q2) *“I believe any initiative that seeks to improve the lives of learners needs our support*. *Also*, *let this not be a ‘policy’ that will exist on paper but it should be implementable*.*”* (Educator in management, Q1)
**Challenges of implementation climate**	
**Inadequate Implementation strategy**	
*“A clear understanding of this programme*, *is it going to be beneficial to them*? *The community but most importantly the learners*.*”* (Educator in management, Q1)	*“If well planned and implemented*, *due dates adhered*, *well managed*.*”* (Educator in management, Q1) *“Parents need to understand the objectives of the study and how the study will help them and their children*.*”* (Educator, Q4)
**Lack of stakeholder involvement**	
*“Lack of involvement by learners*, *teachers and parents*.*”* (Educator, Q3)	*“If all stakeholders can have teamwork or working together for the success of the programme*.*”* (Educator, Q1)
**Lack of consultation or communication**	
*“When you don’t bring the feedback to the people concerned*.*”* (Educator, Q1)	
**Challenges of organisational readiness for change**	
**Non-commitment from educators**	
*“Lack of commitment by stakeholders*.*”* (Educator in management, Q1)	

**Table 3:** Themes identified under educator, learner, parent, system, implementation climate and organisational readiness for change facilitators with illustrative quotations from participants.

**Educator Facilitators**
**Beneficial value for educators**
*“Appreciate that the researcher has chosen our school and it will improve our school*.*”* (Educator in management, Q3) *“I believe in this program even though it might not change the school 100% but at least it would have made a huge difference*.*”* (Educator, Q3) *“Create a message that will resonate with the ideals of educators in terms of them being agents of change in learner' lives*. *Encourage them to see this as being beneficial towards them attaining a healthier learning and teaching environment*.*”* (Educator in management, Q1)
**Learner Facilitators**
**Beneficial value for learners**
*“I think this programme is quite inclusive*, *each and every learner deserves to be part of it*, *they could learn a lot from the programme*.*”* (Educator, Q1) *“I do not see any problems and just see a healthy and a humanity programme*.*”* (Educator, Q3) *“People are sick*, *learners are sick there should be a solution at the end of the day*.*”* (Educator in management, Q1) *“Empowering our community will bring change to our environment and what will be more nicer is when our children speak out and address their concerns about what might be trouble to their community*.*”* (Educator, Q1)
**Parent Facilitators**
**Cascading knowledge**
*“Learners need to cascade the information to the parents*. *As the concerned citizen*, *I think it is time to empower our citizen/community with knowledge*.*”* (Educator, Q1)
**System Facilitators**
**Alignment with curriculum**
*“By ensuring that the programme is in line with CAPS*, *curriculum coverage of those learners*.*”* (Educator in management, Q2)
**Facilitators of implementation climate**
**Educational support for the intervention**
*“Elaborating further about sexual ideas*.*”* (Educator, Q3) *“Through training because when the staff is well trained they are confident to successfully implement the programme*.*”* (Educator in management, Q1)
**Increase in communication with stakeholders**
*“By briefing the staff from time to time about progress made so as to motivate them*.*”* (Educator in management, Q3) *“Linking it with wellness programme that are currently government sanctioned*. *Communicating the results in a manner that does not say*:*' more work for you*, *but better working conditions with learners who are better equipped'*.*”* (Educator in management, Q1)
**Increase in consultation with stakeholders**
*“Discuss issues/challenges that might rise during the research period*.*”* (Educator, Q4) *“If the researcher plans each lesson with at least one member of the staff*, *more ideas may be shared on how these lessons can be implemented*. *Also staff members have more knowledge on how to deliver different activities*, *with experience*, *more can be done*.*”* (Educator, Q1)
**Rewards for implementation**
*“The researcher can bring the rewards to those teachers/staff who are actively involved*.*”* (Educator, Q1) *“Small incentives e*.*g*. *prizes for the learners*.*”* (Educator, Q4)
**Presence of researcher during implementation**
*“To either present the lesson or be with the educator when lesson is given*.*”* (Educator in management, Q4)
**Facilitators of organisational readiness for change**
**Motivation for implementation**
*“The researcher should motivate*, *encourage the staff to implement the programme and show the positive impact the programme will have to the school*.*”* (Educator in management, Q3)
**Commitment for implementation**
*“The commitment of parents and teachers and the learners*. *Parents need to understand the objectives of the study and how the study will help them and their children*.*”* (Educator, Q4)
**Determination to implement intervention**
*“Determination from all that are involved in the program i*.*e*. *parents*, *children*, *teachers and the researcher herself*.*”* (Educator, Q4)
**Encourage teamwork and co-operation**
*“Teachers to implement the program 100%*.*”*(Educator, Q4)

Table 4 shows the overall ICS median score of 3.6 (IQR 3.2–4.1), which was considered acceptable for implementation climate, as 3.2 was chosen as the cut-off point for positive implementation climate. The overall median scale scores were all higher than the cut-off of 3.2.

**Table 4:** Summary statistics for the Implementation Climate Scale.

ICS Scales and Subscales	Median	Interquartile range	Cronbach’s alpha α
**ICS total**	**3.6**	**3.2–4.1**	**0.81**
***Focus of programme***	**3.9**	**3.0–4.2**	0.93
C1. Need for health programmes at schools	4.0	4.0–5.0	
C2. Communication with staff about programme	4.0	3.0–5.0	
C3. Consultation with staff about programme	3.5	3.0–4.0	
C4. Understanding of objectives and goals	3.5	3.0–4.0	
C5. Achievable objectives and goals	4.0	3.0–4.0	
C6. Influence of behaviour change in learners	4.0	3.0–5.0	
C7. Influence of behaviour change in parents	4.0	3.0–4.0	
C8. Importance of programme	4.0	3.0–5.0	
C9. Effective programme implementation	4.0	3.0–4.0	
C10. Using the programme is a top priority	4.0	3.0–4.0	
***Educational support for programme***	**4.0**	**3.0–4.5**	0.93
D1. Relevant training and support	4.0	3.0–5.0	
D2. Relevant training material	4.0	3.0–4.0	
***Recognition for programme***	**3.7**	**2.7–4.0**	0.86
E1. Seen as health specialist	4.0	3.0–4.0	
E2. Held in high esteem	4.0	3.0–4.0	
E3. More likely to be promoted	3.0	2.0–4.0	
***Openness of staff for programme***	**3.6**	**3.2–4.2**	0.84
G1. Staff adaptable to change	3.0	3.0–4.0	
G2. Staff flexible to change	3.0	3.0–4.0	
G3. Staff open to new ideas	3.0	3.0–4.0	
G4. Staff concerned about learner health	4.0	4.0–5.0	
***Openness of staff for programme***	**3.6**	**3.2–4.2**	0.84
G1. Staff adaptable to change	3.0	3.0–4.	
G2. Staff flexible to change	3.0	3.0–4.0	
G3. Staff open to new ideas	3.0	3.0–4.0	
G4. Staff concerned about learner health	4.0	4.0–5.0	
G5. Staff concerned about community health	3.0	3.0–5.0	

Table 5 shows the ICS subscales by school characteristic. Statistically significant differences were identified in the focus of the programme, and recognition for the programme, between school, quintile and fee-paying classification. The quintile 4, fee-paying school was the only school that showed significant differences between the focus of programme (p = 0.005) and recognition for programme (p = 0.014) subscale. The overall ICS mean score for the quintile 4 school was below the 3.2 cut-off.

**Table 5:** The Implementation Climate Scale subscales by characteristics of school.

		Focus of programme Mean (SD)	Focus of programme P-value	Educational support for programme Mean(SD)	Educational support for programme P-value	Recognition for programme Mean (SD)	Recognition for programme P-value	Openness for staff of programme Mean (SD)	ICS score	ICS score P-Value
**Overall ICS**		**3.7 (0.8)**		**3.7 (0.9)**		**3.5 (0.9)**		**3.7 (0.6)**	**3.7 (0.7)**	
**School Code**			0.009		0.029		0.024			0.007
	I	4.1 (0.6)		4.1 (0.8)		3.9 (0.7)		3.8 (0.7)	4.0 (0.5)	
	K	3.6 (0.8)		3.6 (0.8)		3.2 (1.0)		3.7 (0.6)	3.6 (0.7)	
	M	3.4 (0.6)		3.0 (0.6)*	0.016	3.5 (0.4)		3.6 (0.5)	3.4 (0.4)	
	W	2.8 (0.8)*	0.005	3.4 (1.3)		2.5 (1.1)*	0.014	3.4 (0.7)	3.0 (0.6)*	0.014
	Z	4.1 (0.7)		4.2 (0.7)		4.0 (0.9)		4.0 (0.5)	4.1 (0.6)	
**Quintile**			0.008		0.044		0.034			0.015
	1	3.8 (0.8)		3.8 (0.8)		3.5 (1.0)		3.8 (0.6)	3.8 (0.7)	
	2	3.4 (0.6)		3.0 (0.6)*	0.016	3.5 (0.4)		3.6 (0.5)	3.4 (0.4)	
	3	4.1 (0.6)		4.1 (0.8)		3.9 (0.7)		3.8 (0.7)	4.0 (0.5)	
	4	2.8 (0.8)*	0.005	3.4 (1.3)		2.5 (1.1)*	0.014	3.4 (0.7)	3.0 (0.6)*	0.014
**Fee paying school**			0.005				0.014			0.014
	Fee paying	2.8 (0.8)*	0.005	3.4 (1.3)		2.5 (1.1)*	0.014	3.4 (0.7)	3.0 (0.6)*	0.014
	Non-fee paying	3.9 (0.7)		3.8 (0.9)		3.7 (0.8)		3.8 (0.6)	3.8 (0.6)	
**Location**										
	Rural	3.7 (0.8)		3.6 (0.8)		3.5 (0.8)		3.8 (0.6)	3.7 (0.6)	
	Urban	3.7 (0.9)		3.8 (1.0)		3.5 (1.1)		3.6 (0.7)	3.7 (0.7)	
**Position**										
	Educator	3.7 (0.8)		3.8 (0.8)		3.7 (0.9)		3.8 (0.7)	3.8 (0.7)	
	Management	3.6 (0.9)		3.5 (1.0)		3.2 (0.9)		3.6 (0.5)	3.6 (0.7)	

* Statistically significant at 0.05

Most educators believed that they would receive the necessary educational support for the programme, except for the quintile 2 school where significant differences (p = 0.016) were noted. The overall ICS mean score for this school was above the 3.2 cut-off.

Table 2 shows three implementation climate needs which, if not addressed, could be barriers related to focus of the programme. These included clear implementation strategy; extensive stakeholder involvement and consultation; and clear communication. Educators required a clear understanding of the programme and its benefits. They recommended that the intervention be clearly planned, managed and implemented, and dates adhered to. They also requested more information about the programme and its implementation and suggested that additional health-specific information be provided to educators. Inadequate consultation and communication with stakeholders and a lack of stakeholder involvement could also impede implementation. A recommendation was to encourage stakeholder involvement by ensuring that study objectives and benefits were clearly understood. Four implementation climate facilitators included: educational support for the intervention; increase in stakeholder communication; increase in stakeholder consultation; and presence of the researcher during implementation.

Although 43% of participants anticipated an increase in workload, 61% indicated that the programme would directly benefit them (Table 6). Most (74%) participants thought the programme would benefit the learners, 61% thought that it would also benefit the parents, and 57% thought that it would also benefit the community. Other suggestions were ‘*capacity building’* and ‘*three parties will benefit if all can participate’*, as additional impacts. Cascading knowledge from learners to their parents was a recognised benefit. Educators believed that when children acquire knowledge, they often pass it on to parents and the community. “*Learners need to cascade the information to the parents*. *As the concerned citizen*, *I think it is time to empower our citizen/community with knowledge”* (Quintile 1 educator). Benefits for educators and learners were anticipated, such as better teaching and learning environments. “*Create a message that will resonate with the ideals of educators in terms of them being agents of change in learner' lives*. *Encourage them to see this as being beneficial towards them attaining a healthier learning and teaching environment”* (Quintile 1 educator) and, “*I think this programme is quite inclusive*, *each and every learner deserves to be part of it”* (Quintile 1 educator).

**Table 6:** Educator perceptions of how programme may affect participants.

Educator perception of impact of the programme	n	%
Increase in workload for teachers	20	43
Increase in monitoring and evaluation for teachers	16	35
Increase in parent interaction	25	54
Reduce in absenteeism at school	16	35
Benefit the learners	34	74
Benefit teachers	28	61
Benefit parents	28	61
Benefit the community	26	57
No impact at all	0	0
Other	2	4

Increased educator workload was a perceived barrier, as were time constraints. Recommendations to facilitate the process were to not have stringent implementation rules and techniques, recruit more educators, have additional support for educators, and make the benefits of the programme known. “*The problem that I foresee is time and workload but if managed properly it will bring out the beauty of diverse cultures*” (Quintile 4 educator).

Rewards for implementation were identified by educators in the quintile 4, fee-paying school as a necessity. Educators requested to be financially remunerated for the implementation and saw this as a motivation. Rewards were seen as motivators by others too, but this included learner rewards rather than direct remuneration as identified in the quintile 4 school

Table 7 shows the overall ORIC median score of 4.3 (IQR 3.8–4.9). Subscale content was evaluated in relation to the two theoretically defined constructs, change commitment and change efficacy (4.2 and 4.3, respectively). The ORIC median score and the individual subscale scores were all higher than the average score of 4 and were considered to be acceptable.

**Table 7:** Summary statistics for the ORIC scales and subscales.

	Median	Interquartile range	Cronbach’s alpha α
**ORIC total**	**4.3**	**3.8–4.9**	**0.93**
***Change Commitment***	**4.2**	**3.6–5.0**	0.94
H2. Want to implement change	4.0	4.5–5.0	
H3. Do whatever it takes to implement programme	4.0	3.0–5.0	
H4. Determined to implement programme	4.0	4.0–5.0	
H5. Motivated to implement programme	5.0	4.0–5.0	
H6. Committed to implement programme	4.0	4.0–5.0	
***Change Efficacy***	**4.3**	**4.0–4.9**	0.89
H1. Keep momentum going	5.0	4.0–5.0	
H7. Coordinate tasks for smooth implementation	4.5	4.0–5.0	
H8. Keep track of progress during implementation	5.0	4.0–5.0	
H9. Handle implementation challenges that arise	4.0	4.0–5.0	
H10. Get the learners invested in programme	4.0	4.0–5.0	
H11. Support learners/parents as they adjust to change	5.0	4.0–5.0	
H12. Manage the politics of implementation	4.0	3.0–5.0	

As shown in Table 8, significant differences were noted in the overall ORIC score between schools, quintiles and fee-paying schools. The quintile 4 fee-paying school had the lowest overall ORIC score of 3.4. Although no significant differences were observed in change efficacy, significant differences were observed in change commitment (p = 0.007), which was again seen at the quintile 4 fee-paying school.

**Table 8:** Individual-level and team-level ORIC subscale correlation matrix.

		Change commitment Mean (SD)	Change commit-ment P-value	Change efficacy Mean (SD)	ORIC score Mean (SD)	ORIC Score P-value
**Overall ORIC score**		**4.2 (0.8)**		**4.3 (0.7)**	**4.2 (0.7)**	
**School**			0.009			0.018
	I	4.4 (0.6)		4.5 (0.5)	4.5 (0.5)	
	K	4.1 (0.8)		4.1 (0.7)	4.1 (0.8)	
	M	3.9 (0.7)		4.1 (0.5)	4.0 (0.4)	
	W	3.2 (1.0)*	0.007	3.7 (1.0)	3.4 (1.1)*	0.019
	Z	4.9 (0.2)		4.7 (0.3)	4.8 (0.2)	
**Quintile**			0.020			0.034
	1	4.5 (0.7)		4.4 (0.6)	4.4 (0.7)	
	2	3.9 (0.7)		4.1 (0.5)	4.0 (0.6)	
	3	4.4 (0.6)		4.5 (0.5)	4.5 (0.5)	
	4	3.2 (1.0)*	0.007	3.7 (1.0)	3.4 (1.1)*	0.019
**Fee paying**						0.019
	Fee paying	3.2 (1.0)*	0.007	3.7 (1.0)	3.4 (1.1)*	0.019
	Non-fee paying	4.2 (0.7)		4.4 (0.5)	4.4 (0.6)	
**Location**						
	Rural	4.2 (0.8)		4.3 (0.7)	4.3 (0.7)	
	Urban	4.0 (0.9)		4.3 (0.7)	4.1 (0.9)	
**Position**						
	Educator	4.2 (0.8)		4.4 (0.6)	4.3 (0.7)	
	Management	4.0 (0.9)		4.1 (0.7)	4.1 (0.8)	

* Statistically significant at 0.05

Educators from the quintile 4 fee-paying school were least receptive to the programme. They saw no benefit in assisting with ‘*yet more research*’ that they perceived did not benefit them directly. The lack of financial incentives for implementing the intervention was seen as a possible barrier by both management and some educators. This lack of interest differed from other schools where staff were welcoming and encouraging. A quintile 3 educator in management stated, “*Appreciate that the researcher has chosen our school and it will improve our school*” and “*this program even though it might not change the school 100% but at least it would have made a huge difference*”.

Table 2 shows that non-commitment from educators was the only ORIC barrier that was identified. Table 4 presents four ORIC facilitators that were identified: motivation, commitment, determination, and encouraging teamwork and co-operation.

Other barriers and facilitators that did not fit into the implementation climate and ORIC constructs were also identified. Parent non-participation was perceived as a barrier where some parents may have no interest, especially “*younger parents who did not take things seriously*”. Additionally, general school communication with parents was via the learner, which was inefficient. “*The only challenge that I see*, *about co-operation from parents*, *in terms of information getting to them*. *We communicate with parents through the learners who do not get information to parents on time”* (Quintile 1 management). Educators recommended that parents needed to become willing participants and be open to the programme, which could be achieved by engaging them and providing a clear understanding of the programme.

Educators felt that implementation could be impeded if parents and learners believed that the intervention was inappropriate or unacceptable. They believed that the majority of parents would not be willing to participate or involve their children; that parents may not understand and accept the programme; and that it was difficult to talk about your health problems. *“Some parents might not be free to communicate certain aspects of life with their kids at this age”*(Quintile 1 management); and *“Our parents* [some] *can retard the program as they will be scared that someone will know their* [HIV] *status”* (Quintile 1 educator). A quintile 1 educator claimed that many learners at that school were orphans and lived at an orphanage or with other family members; and perceived that their parents had lost their lives due to HIV/AIDS. Another quintile 2 educator said that the daily meals received by learners were sometimes the only meal received, and they often took it home to share with family. It was also recommended that the researcher make home visits to the learners’ houses to appreciate the environment in which learners live.

Educators felt that learners are from different cultures and societies and not ensuring that the intervention was culturally suited might limit the intervention. *“Culture is a big problem (kids not allowed to question elders about personal matters)”* (Quintile 1 educator); *“Culturally*, *parents will not feel free to communicate confidential information to their children”* (Quintile 2 educator); *“If some learners confide to you*, *how are you going to deal with that*?…s*ensitivity when dealing with learners because some come from families that are directly affected by some of the diseases mentioned*.*”* (Quintile 3 educator).

Consultation and two-way communication were seen as facilitators of implementation. Educators requested feedback at each stage, follow-ups, and results at the end of the study. They said that they were keen on fixing any issues or challenges that might arise during the implementation. “*If the researcher plans each lesson with at least one member of the staff*, *more ideas may be shared on how these lessons can be implemented*. *Also*, *staff members have more knowledge on how to deliver different activities*, *with experience*, *more can be done*” (Quintile 2 educator).

Educators perceived that policy implications and reluctance from the government in the application of any positive results would create a barrier in terms of whether the intervention would be recognised and scaled-up. A quintile 1 educator in management recommended that “*ensuring that the programme is in line with CAPS curriculum*” would facilitate implementation and sustainability. Educators’ recommendations were to incorporate any positive outcomes into departmental policies, taking into consideration that resource availability might be another challenge.

## Discussion

This study aimed to evaluate the level of organisational readiness and the associated barriers and facilitators to the implementation of a *The CIrCLE of Life Initiative* within South African primary schools. The level of OR was based on overall scores from the ORIC and implementation climate constructs. The acceptable results indicate the degree of fit between the significance and values attached to the intervention by educators and how the intervention would be received by the target group (parents and learners). The assessment also identified fundamental facilitating factors, needs, and the role of context, which prompted further planning and improvement to the intervention prior to implementation. The improvements could influence implementation effectiveness.

Acceptable results from the ORIC suggested that educators collectively valued the change highly enough to commit to its implementation. When ORIC is high, educators may more skilfully and persistently action the health intervention and may demonstrate a more consistent, high-quality use of the intervention [2], indicating that greater readiness may lead to more effective implementation. Educators from four of the five schools saw a need for and welcomed the intervention. Although the overall ORIC scores were acceptable, the quintile 4, fee-paying school, differed from the other schools, having the lowest score. This indicated an expectation that educators may resist initiating change, put less effort into implementation, persevere less in the face of implementation challenges, and exhibit compliant intervention utilisation [2].

Change efficacy was high, indicating that educators had a positive assessment of organisational implementation capability and that they could collectively implement the organisational change. Barriers such as non-commitment of educators could result in poor implementation of the programme. Their recommendations to address non-commitment were to increase motivation, determination, and encourage teamwork and co-operation.

The findings show that the two facets of change commitment and change efficacy could be conceptually interrelated. While no significant differences were observed in change efficacy, significant differences were observed in change commitment in the quintile 4 school. The educators at this school were confident that they could implement the change successfully, yet showed no commitment to do so. Organisational readiness is likely to be highest when organisational members not only feel confident that they can implement an organisational change, but also want to do so [2].

The results of the implementation climate subscale focus of the programme demonstrated that the motivation for the health programme, goals and objectives, and realisation for change and the benefits thereof, were well-comprehended by educators. The perceived degree of fit between the significance and values attached to the intervention by educators, and how these would be received by the target group, was also a beneficial finding. When implementation climate is high in the organisation, employees perceive the organisation values the intervention and is supportive in implementing it. It has been proposed that such perceptions lead to increased levels of attention and motivation for intervention implementation, ultimately enhancing intervention outcomes [12,32].

Educators believed that their direct involvement in a project of this nature was a way to be recognised, thereby increasing their chances of promotion. Management was not of the same opinion. Stakeholders who are members of the organisation either use an innovation directly (e.g. front-line staff) or support an innovation’s use (e.g. management) [10]. Perceptions of organisational incentives and rewards, which include extrinsic incentives (goal-sharing awards, performance reviews, promotions, and less tangible incentives such as increased stature or respect) varied between educators and management. A sense of respect and professionalism may increase job satisfaction and teamwork, and help teachers feel equipped to undertake new curricula, supported when undertaking new initiatives, and part of a broader mission of change at the school [23].

The overall ICS score for the quintile 4 fee-paying school was the only score that was below the cut-off, and showed significant differences across two of the subscales: focus of programme and recognition for programme. Perceived barriers related to implementation climate included an inadequate implementation strategy and the lack of involvement, consultation and communication with relevant stakeholders. Recommendations, related to implementation climate, to facilitate implementation included more educational support for the intervention, the presence of the researcher during implementation, rewards/incentives for educators and learners, and additional communication and consultation with all stakeholders. Stakeholder consultation influences the nature and direction of the intervention. Such dialogue not only fosters more commitment but gives implementers a sense of ownership [33].

From the identified key barriers and facilitators identified, it became apparent that the health intervention needed to be a fit with existing workflows and educational systems. One of the main barriers for educators was the increased workload. Their recommendations were to motivate the staff, apply non-stringent implementation rules and techniques, recruit more educators, and provide additional support. Alignment of the intervention with the curricula and syllabus would also facilitate their workflow. One of the identified system challenges was the timing and scheduling of components of the intervention. Educators perceived that the timing and length of the programme might impede implementation if not linked to educators’/schools’ specific timelines.

Innovation-values fit, such as intervention appropriateness and acceptability, and sensitivity to HIV were identified. Dealing with personal health-related matters such as HIV is a sensitive issue, more so because of the associated stigma. To address the issue of appropriateness and acceptability, to not hinder implementation, the implementers needed to deal with these contextual issues in a sensitive manner which required incorporation into the educator training modules. Not only did the intervention need to be culturally suited, appropriate and acceptable, but educators needed to be trained on emotional and sensitivity aspects–an area in which educators lacked knowledge and confidence to deliver. Educators believed that both initial and ongoing training was paramount to build self-efficacy for project implementation.

Weiner (2011) [32], suggests that OR measures should be descriptive and not evaluative. However, Klein and Sorra (2008) [34] advise that the descriptive-evaluative distinction could be viewed as a continuum rather than a dichotomy, with an inclination towards the descriptive side of the continuum. This study utilised both quantitative and qualitative approaches which had several merits. The qualitative approach helped to measure organisational levels as a useful benchmark on the continuum, and enabled a more precise quantitative estimation of relationships between constructs [11]. The qualitative data offered insight into the nuances and influences of the constructs [11]. The qualitative data were, therefore, of greater value in advancing implementation, encouraging a proactive approach. The alignment of the quantitative findings with the qualitative theory proved to be valuable. Strategies need to mitigate factors that serve as barriers to support implementation.

This transdisciplinary intervention presents unique challenges as it requires educators to implement a health intervention across geographical boundaries and school settings. The collaboration required between the Departments of Health and Education necessitated that educators be open to diverse viewpoints, mutual deliberation, and problem-solving for the effort to be effective [35]. Educator training is a planned component of the pre-implementation phase. The findings indicated, however, that the planned training required more in-depth guidance than what was originally planned, not only with training in implementation, health concepts and implementation strategy, but also fundamental health concepts covered in the current school curriculum. This weakness had to be resolved to ensure that educators were more competent in teaching health and answering questions. Staff development provides the necessary support to update educators’ pedagogical and content knowledge, as well as enhances their competence and confidence levels [33].

Educators also requested the presence of the researcher during class lessons for reassurance and support. Vicarious learning strategies (e.g. site visits) are useful for supplying organisational members with accurate information about task demands and situational factors affecting implementation, [2] and had to be incorporated into the implementation. Educators also advised that implementation strategies that were not well-defined and aptly communicated could pose barriers. The implementation process will be more effective if the roles of educators were made more clearer [33].

### Strengths and limitations

The results of this study add to the limited body of evidence on implementation climate and OR for change. Two diverse OR constructs with differing scales and goals were used in the evaluation; however, their similar design and compatible features permitted a congruent union of the two constructs. Although the ICS and ORIC tools were adapted to suit the study context and design, the high reliability in consistency, shown by the Cronbach alpha scales, was an additional strength of the study.

This study found that although the quantitative evaluations from both constructs were beneficial, the tools still required an additional qualitative assessment. Facilitators and barriers identified through this study were not derived from the original quantitative construct but additional qualitative research. The constructs also lack insight from other implementation science theories such as culture, appropriateness and acceptability. Such issues, in this study, were identified from the qualitative data. Improvement of the constructs should, therefore, be continued. In addition, certain scales, e.g. the rewards for EBP, merit further theoretical and empirical attention.

Another limitation of the study was the small sample size; although it was large enough to measure OR in the study, it restricted additional statistical analysis.

## Conclusion

This study married organisational readiness to implement change and implementation climate constructs to ascertain underlying organisational conditions in schools before the implementation of a transdisciplinary health promotion and prevention intervention: *The CIrCLE of Life Initiative*. The results provided useful insights into potential areas of need and challenges that could hinder effective implementation allowing for further improvements to be made to the intervention and the implementation strategy.

The results demonstrate the necessity for assessing OR prior to any intervention implementation. Effective evaluation of OR could reduce the number of change efforts in the organisation and prevent undesired consequences of the intervention implementation. It is incumbent on researchers who contribute to the process of research and practical application to address organisational factors as a criterion for enhanced implementation success.

## Supporting information

S1 File This file includes a manuscript that is currently under review, titled, Development of a theoretical and evidence-based practice to address the double burden of disease through a pre-adolescent transdisciplinary intervention for inter-generational outcomes: an intervention mapping approach. (DOCX)

S2 File This file presents the organisational readiness questionnaire that was used to collect data in the study. (DOCX)

S1 Table Standards for Reporting Implementation Studies: The StaRI checklist. This table presents a checklist with details regarding the implementation strategy that was used during the study.
(DOCX)

## References

[1] Scaccia Jonathan P., Cook Brittany S., Lamont Andrea, Wandersman Abraham, Castellow Jennifer and JK. A practical imlementation science heuristic for organisational readiness: R = MC2. J Community Psychol. 2015;43: 484–501. 10.1002/jcop.21698 26668443 PMC4676714

[2] Weiner BJ. A theory of organizational readiness for change. Implement Sci. 2009;4 10.1186/1748-5908-4-67 19840381 PMC2770024

[3] Stokols D. Toward a science of transdisciplinary action research. Am J Community Psychol. 2006;38: 63–77. 10.1007/s10464-006-9060-5 16791514

[4] Pillay-van Wyk V, Msemburi W, Laubscher R, Dorrington RE, Groenewald P, Glass T, et al Mortality trends and differentials in South Africa from 1997 to 2012: second National Burden of Disease Study. Lancet Glob Heal. 2016;4: e642–e653. 10.1016/S2214-109X(16)30113-9 27539806

[5] Devanathan R, Esterhuizen TM, Govender RD. Overweight and obesity amongst Black women in Durban, KwaZulu-Natal: A “disease” of perception in an area of high HIV prevalence. African J Prim Heal Care Fam Med. 2013;5: 1–7. 10.4102/phcfm.v5i1.450

[6] Averett SL, Stacey N, Wang Y. Decomposing race and gender differences in underweight and obesity in South Africa. Econ Hum Biol. 2014;15: 23–40. 10.1016/j.ehb.2014.05.003 25434513

[7] Damerell P, Howe C, Milner-Gulland EJ. Child-orientated environmental education influences adult knowledge and household behaviour. Environ Res Lett. 2013;8: 015016 10.1088/1748-9326/8/1/015016

[8] NIH Fogarty International Center. Implementation science information and resources. 2017 [cited 14 Sep 2017]. Available: https://www.fic.nih.gov/researchtopics/pages/implementationscience.aspx

[9] Grimshaw JM, Eccles MP, Lavis JN, Hill SJ, Squires JE. Knowledge translation of research findings. Implement Sci. 2012;7: 1 10.1186/1748-5908-7-50 22651257 PMC3462671

[10] Klein KJ, Sorra JS. The Challenge of Innovation Implementation. Acad Manag Rev. 2008;21: 1055–1080.

[11] Weiner B, Lewis M, Linnan L. Using organization theory to understand the determinants of effective implementation of worksite health promotion programs. Health Educ Res. 2009;24: 292–305. 10.1093/her/cyn019 18469319

[12] Ehrhart MG, Torres EM, Wright LA, Martinez SY, Aarons GA. Validating the Implementation Climate Scale (ICS) in child welfare organizations. Child Abus Negl. 2016;53: 17–26. 10.1016/j.chiabu.2015.10.017 26563643 PMC4818155

[13] Storkholm MH, Mazzocato P, Tessma MK, Savage C. Assessing the reliability and validity of the Danish version of Organizational Readiness for Implementing Change (ORIC). Implement Sci. 2018;13: 1–7. 10.1186/s13012-017-0699-0 29871691 PMC5989337

[14] Shea CM, Jacobs SR, Esserman DA, Bruce K, Weiner BJ. Organizational readiness for implementing change: a psychometric assessment of a new measure. Implement Sci. 2014;9: 7 10.1186/1748-5908-9-7 24410955 PMC3904699

[15] Ehrhart MG, Aarons GA, Farahnak LR. Assessing the organizational context for EBP implementation: the development and validity testing of the Implementation Climate Scale (ICS). Implement Sci. 2014;9: 157 10.1186/s13012-014-0157-1 25338781 PMC4210525

[16] Lyon AR, Cook CR, Brown EC, Locke J, Davis C, Ehrhart M, et al Assessing organizational implementation context in the education sector: confirmatory factor analysis of measures of implementation leadership, climate, and citizenship. J Implement Sci. 2018;13: 1–14. 10.1186/s13012-017-0705-6 29310673 PMC5759223

[17] Hamilton S, McLaren S, Mulhall A. Assessing organisational readiness for change: Use of diagnostic analysis prior to the implementation of a multidisciplinary assessment for acute stroke care. Implement Sci. 2007;2: 1–11. 10.1186/1748-5908-2-1 17629929 PMC1948015

[18] Hagedorn HJ, Heideman PW. The relationship between baseline Organizational Readiness to Change Assessment subscale scores and implementation of hepatitis. Implement Sci. 2010;5 Available: http://www.biomedcentral.com/content/pdf/1748-5908-5-46.pdf 10.1186/1748-5908-5-46 PMC2902416 20546584

[19] Nilsen P, Wallerstedt B, Behm L, Ahlström G. Towards evidence-based palliative care in nursing homes in Sweden: A qualitative study informed by the organizational readiness to change theory. Implement Sci. 2018;13: 1–12. 10.1186/s13012-017-0699-0 29301543 PMC5753464

[20] Lynch D, Smith R, Yeigh T, Provost S. A study into “organisational readiness” and its impacts on school improvement. Int J Educ Manag. 2019;33: 393–408. 10.1108/IJEM-07-2017-0181

[21] Oterkiil C, Ertesvåg SK. Schools’ readiness and capacity to improve matter. Educ Inq. 2014;3: 71–92. 10.3402/edui.v3i1.22014

[22] Gugglberger L, Dür W. Capacity building in and for health promoting schools: Results from a qualitative study. Health Policy (New York). 2011;101: 37–43. 10.1016/j.healthpol.2010.08.019 20855125

[23] Gregory A, Henry DB, Schoeny ME, Eron L, Guerra N, Henry D, et al School climate and implementation of a preventive intervention. Am J Community Psychol. 2007;40: 250–260. 10.1007/s10464-007-9142-z 17917806

[24] Naylor PJ, Nettlefold L, Race D, Hoy C, Ashe MC, Wharf Higgins J, et al Implementation of school based physical activity interventions: A systematic review. Prev Med (Baltim). 2015;72: 95–115. 10.1016/j.ypmed.2014.12.034 25575800

[25] Schaap R, Bessems K, Otten R, Kremers S, van Nassau F. Measuring implementation fidelity of school-based obesity prevention programmes: A systematic review. Int J Behav Nutr Phys Act. 2018;15 10.1186/s12966-018-0709-x 30103764 PMC6088402

[26] Zhang W, Creswell J. The use of mixing procedure of mixed methods in health services research. Med Care. 2013;51: 51–57. 10.1097/MLR.0b013e31824642fd 23860333

[27] The Government Gazette. The national norms and standards for school funding. Pretoria; 2006.

[28] Gardiner M. Priority Areas for Educational Attention in South Africa by Michael Gardiner. Johannesburg; 2006.

[29] Pinnock H, Barwick M, Carpenter CR, Eldridge S, Grandes G, Griffiths CJ, et al Standards for Reporting Implementation Studies (StaRI) Statement. BMJ. 2017;356 10.1136/bmj.i6795 28264797 PMC5421438

[30] Erlingsson C, Brysiewicz P. A hands-on guide to doing content analysis. African J Emerg Med. 2017;7: 93–99. 10.1016/j.afjem.2017.08.001 30456117 PMC6234169

[31] Braun V, Clarke V. Using thematic analysis in psychology. Qual Res Psychol. 2006;3: 77–101. 10.1191/1478088706qp063oa

[32] Weiner BJ, Belden CM, Bergmire DM, Johnston M. The meaning and measurement of implementation climate. Implement Sci. 2011;6: 1–12. 10.1186/1748-5908-6-1 21781328 PMC3224582

[33] Mangwaya E, Blignaut S, Pillay SK. The readiness of schools in Zimbabwe for the implementation of early childhood education. South African J Educ. 2016;36: 1–8. 10.15700/saje.v36n1a792

[34] Klein K, Conn A, Smith D, Sorra J. Is everyone in agreement? An exploration of within-group agreement in employee perceptions of the work environment. J Appl Soc Psychol. 2001;86: 11302231. 10.1037/0021-9010.86.1.3 11302231

[35] Harper GW, Neubauer LC, Francisco VT. Transdisciplinary Research and Evaluation for Community Health Initiatives. NIH Public Access. 2010;9: 328–337. 10.1177/1524839908325334 18936267 PMC2836480

