# Supplementary material for: Educators’ perceptions of organisational readiness for implementation of a pre-adolescent transdisciplinary school health intervention for inter-generational outcomes
Source: PLoS One. 2020 Jan 8;15(1):e0227519. doi: 10.1371/journal.pone.0227519 (PMC6948754; doi:10.1371/journal.pone.0227519)
Supplement: S1 File — (DOCX) [file pone.0227519.s001.docx]

**Development of a theoretical and evidence-based practice to address the double burden of disease through a pre-adolescent transdisciplinary intervention for inter-generational outcomes: an intervention mapping approach**

Keshni Arthur^1*^, Nicola Christofides^1,2^ and Gill Nelson^1,3^

________________________________________________________

*Correspondence: Kesh@Karthurhmc.co.za

^1^School of public health, University of the Witwatersrand,

27 St Andrews Road, Parktown, 2193, Johannesburg, South Africa

Full list of author information is available at the end of the article

**Abstract**

**Background:** South Africa is in the midst of a health transition that is characterised by a double burden of communicable and non-communicable disease. With the limited success of conventional approaches in addressing the burden of disease, utilising transdisciplinary approaches that engage the education sector in disease prevention, could provide holistic healthcare solutions. The study aimed to develop a combined HIV and obesity health intervention that could support knowledge transfer and translation directly to pre-adolescent learners and indirectly to their parents, for immediate- and long-term benefits.

**Methods:** The intervention mapping protocol was used in the design and development of the intervention. As part of the formative study, a scoping review and needs assessment was performed.

**Results:** The scoping review shortlisted 12 evidence-based practices, published from 2007 to 2017, of which selected elements were included in the intervention design. The needs assessment survey investigated educators’ perceptions about the health status of learners. Educators perceived that the health and lifestyle of a learner affect learning in the classroom and saw a need for school-based health programmes. The formative research facilitated the conceptualisation of a logic model of the problem. Performance objectives, determinants of behaviour change, and required behavioural action to modify behavioural change, were identified. Selected theory-based methods were translated into practical strategies. An integrated communicable disease (HIV) and non-communicable disease (obesity) transdisciplinary intervention was developed, along with accompanying resources.

**Conclusion:** The development of *The CIrCLE of Life Initiative* was grounded on proficiency from both theoretical- and evidence-based practices. Theoretical practices such as the taxonomies of behaviour change, amalgamated with the application of the intervention mapping heuristic were assimilated into the design and development of the intervention. Adaption of evidence-based practices ensured that the intervention was a fit with the target population, culture, and context. Stakeholder engagement and collaboration supported the process, with the provision of beneficial parameters that resulted in the preparation of knowledge and skill-enhancing practical strategies for dissemination, adoption, implementation and sustainability.

**Keywords:** Intervention development, Transdisciplinary intervention, Non-communicable disease, Communicable disease, HIV, Obesity, Implementation science

**Background**

The double burden of communicable disease (CD) and non-communicable disease (NCD) is an intricate global health concern (1). In sub-Saharan Africa, the double burden of disease affects an already fragile health system, resulting in further adverse impacts on the health of people (2). Upstream social determinants that influence behaviours that lead to CD and NCD are complex and often intertwined. In a socio-ecological model (3) social determinants include individual-level drivers such as knowledge, attitudes, and educational attainment; community-level drivers such as the level of socioeconomic status, gender norms, urbanisation, the built environment, and the food system; and macro-level drivers such as law and policy (4).

South Africa, like many other developing countries, is in the midst of a health transition that is characterised by high levels of both CDs and NCDs. While South Africa continues to deal with the challenge of CDs, such as human immunodeficiency virus (HIV), acquired immune deficiency syndrome (AIDS) and tuberculosis (TB) (5), the upsurge in obesity (6) and accompanying NCDs (7), such as cardiovascular disease and diabetes, present a novel set of problems. For this study, diseases that were the focus of the intervention were limited to HIV and obesity as these conditions are prevalent in South Africa (6–8).

Body Mass Index (BMI) is a weight-for-height index that is used to classify underweight, overweight or obese (9). Based on BMI score, about two-thirds (68%) of South African women (15 years and older) are overweight (BMI ≥ 25 kg/m2) or obese (BMI ≥ 30 kg/m2), and 30% are in the normal weight range, whereas one-third (31%) of men (15 years and older) are overweight or obese, and 59% have BMIs in the normal range (10). These statistics highlight the obesity problem in South African adult populations and the higher risk in females. Based on weight-for-height, 13% of South African children under the age of five years are overweight; a prevalence of more than twice the global average of 6.1% (10). Obesity results from an imbalance between consumption and expenditure of energy and risk factors include energy-dense diets, physical inactivity, poor early childhood feeding practices, low level of education, lack of knowledge, genetics, cultural factors and high-stress levels (11–14).

HIV affects an estimated 12.6% of the total South African population (15). Approximately one-fifth of South African women in their reproductive ages (15-49 years) are HIV positive (15). Risky sexual behaviours that lead to HIV infection among young people include early sexual debut, multiple partners, forced sex and lack of condom use (16).

While much of the recent attention on CDs such as HIV/AIDS and TB has been on better access to treatment and improved adherence, on-going health promotion and prevention efforts that address social determinants remain critical (7,17,18). Communicable diseases affect both individuals and communities, and control methods should be directed at both levels. Similarly, addressing behavioural and social determinants of NCDs early in life could prevent the onset of one or many NCDs (19,20). Non-communicable diseases are associated with behavioural risk factors such as tobacco use, alcohol consumption, unhealthy diet (including high fat, sugar and sodium intake), physical inactivity and obesity (19).

Low and middle-income countries have tackled CDs using various preventive interventions, and an increasing number have addressed NCDs. However, these are often small scale or pilot programmes with a limited effect at the population level (5,21–24). Early interventions are less frequent and are exacerbated by limited access to holistic and comprehensive services and resources. With the limited success of conventional approaches in addressing the complexities of the double burden of disease, interventions using transdisciplinary approaches to prevent such disease could provide holistic healthcare solutions across disciplinary boundaries (25). Engaging the education sector, for example, as participants in health programmes, could offer a dynamic strategy across the social spectrum because of their existing organisational, social and communication structures (26).

Current interventions are targeted at adult populations, often ignoring pre- and adolescent populations that might influence behaviours at an early stage to prevent disease outcomes. (27,28). Pre-adolescence (9-12 years old) could serve as an important developmental period for prevention efforts (29). Providing pre-adolescents with age- and culture-appropriate skills may serve to guide the developmental trajectory of future risky behaviours toward safer practices. Implementing interventions at an early stage could also stimulate interest and influence health behaviours amongst their parents (upon whom children are most dependent for their health needs and well-being) (30). The term ‘parent’ is considered to be synonymous with ‘guardian’ in this paper.

South African urban and rural schools have a complicated history due to Apartheid systems and practices. Conditions of schools in rural compared to urban areas have many shortcomings due to circumstances of poverty and under-development (31). In South Africa, schools (urban and rural) are categorised into five groups or quintiles (32), based on the relative wealth of their surrounding communities. Schools in the most impoverished communities are classified as Quintile 1 and schools serving the wealthiest communities, as Quintile 5. Quintiles 1, 2 and 3 schools do not charge fees. The Government subsidises schools accordingly; Quintile 1 schools receive the highest allocation per learner and Quintile 5 schools receive the lowest (32).

A study was designed to develop a health intervention that could support knowledge transfer and translation directly to learners and indirectly to their parents. This paper describes the development of a theoretical and evidence-based NCD (obesity) and CD (HIV) transdisciplinary intervention designed for pre-adolescent learners, for immediate- and long-term benefits to both themselves and their parents.

**Methods**

The Intervention Mapping (IM) (33) approach was used in the design and development of the intervention. The emphasis of the intervention was on changing both environmental and behavioural factors that impact HIV and obesity outcomes, in both learners and their parents. Intervention mapping has been recommended as a tool in the development and application of theories (16,26,34,35). It is a stepwise technique for systematically applying evidence-based practice (EBP), relevant theories and additional literature in the development and implementation of interventions to encourage behaviour change. The IM tool comprises six steps. This paper focuses on the development of the intervention (steps 1 to 4) and on the peripheral conditions in planning for the implementation of the intervention (step 5 and 6).

Step 1 comprised a scoping review and a needs assessment which helped conceptualise the logic model of the problem. Step 2 was the formulation of programme objectives. This led to the development of various performance objectives at both the individual (learner) and interpersonal (parents) levels fitting the socio-ecological model. Personal and external determinants were identified and then matched with the performance objectives to develop matrices of specific change objectives. In step 3, theory-based methods most certain to influence changes in the selected determinants and the conditions under which the methods were most probable to be effective, were identified. The theory-based methods were subsequently translated into practical strategies that corresponded with the change objectives from the matrices. Step 4 comprised a description of the scope and sequence of the components of the intervention. Step 5 supported the design of the implementation and adoption plan which defined implementation objectives, methods and strategies. Last, Step 6 entailed the development of an evaluation plan.

**The scoping review (Step 1)**

The scoping review mapped the literature on school-based health interventions designed to reduce the risk of obesity and HIV in pre-adolescents and their parents. The purpose of the review was to identify, describe and shortlist relevant EBPs.

The five-stage methodological process outlined by Arksey and O’Malley (2005) (36) was used to conduct the review. The five stages were undertaken to determine the research question, identify relevant literature, select appropriate studies, chart the data, and collate, summarise and report results. A Preferred Reporting Items for Systematic reviews and Meta-Analyses extension for Scoping Reviews (PRISMA-ScR) Checklist (37) was used to further improve the completeness and replicability of reporting of this review and is available in Additional file 1.

In selecting studies, the search parameters were limited to the following inclusion criteria: EBP of HIV or obesity school-based intervention studies that reported effectiveness as an outcome, including both pre-adolescent/adolescent learners and their parents, studies written in English, and published from 2007 to 2017. Grey literature, non-English documents, and those that mentioned ‘intervention’ in a framework without further elaboration of the interventions or intervention components, were excluded. The PRISMA flow diagram (38) was used to report the identification, screening and inclusion process and is available in Additional file 2.

The following electronic databases were searched from January to February 2018: MEDLINE (via PubMed) and Cochrane Database (via The Cochrane Library). The search strategy included the keywords ‘school-based’, ‘HIV intervention’, ‘obesity intervention’, ‘parent’ and ‘adolescent’. The search terms are presented in Additional file 3.

References were imported into an EndNote Reference Manager library. Using EndNote, duplicates were removed, full-text papers were obtained, and records were stored and managed. Tables were designed to summarise the identified studies for collation and reporting.

**Formative research: needs assessment (Step 1)**

A needs assessment was conducted to assess the HIV and obesity health status at selected schools; the environmental conditions and behaviour/s associated with disease, and the desired programme outcomes. The schools are five Government-run primary schools in a district located in Gauteng province, South Africa. Two, are classified as a quintile 1 schools, and the other three represent quintile 2, 3 and 4 schools. Three of the schools are in a rural area; two are located in an urban area. The sizes of the schools range from 650 – 1100 learners with a teaching complement of 25 – 30 educators. There are approximately 100 Grade six learners in each school. Mining is the main industry in the district and diseases such as HIV/AIDS and TB are prevalent.

As part of the primary data collection, an information session was held with the management (principals, deputy principals and heads of department) and educators of the schools, who would potentially interact with the programme. Educators answered a self-administered questionnaire that explored the health status of learners at their schools. A discussion with participants followed, and the researcher documented observations and feedback.

The Human Research Ethics Committee of the Health Sciences Faculty, University of the Witwatersrand approved the study (clearance certificate no. M180220). Participation was voluntary, and participants signed consent to participate.

Data from the questionnaires were captured using RedCap and were then exported into Stata 14 for statistical analysis. Proportions or mean scores with standard deviations, or median scores with interquartile ranges, were calculated for educator perceptions of the health status of the learners and the community. All documented details and observations were transcribed with the permission of the participants.

The Template for Intervention Description and Replication (TIDieR) checklist (39) was used to further improve the completeness and replicability of reporting of this intervention and is available in Additional file 4.

**Results**

**Step 1: Formative research and logic model of the problem**

The scoping review

The scoping review, which identified EBPs on school-based HIV and obesity interventions, informed the needs assessment and provided a base for the intervention design.

**Table 1: Descriptive summary of studies included in scoping review**

| Classification scheme | Year of study | Country | Study design | Target population | Focus of Intervention | Primary outcome | Secondary outcome | Result |
| --- | --- | --- | --- | --- | --- | --- | --- | --- |
| Adab et al. (49) | 2017 | United Kingdom | RCT | Pre-adolescents and parents | Obesity | BMI | Further anthropometric, dietary, physical activity, and psychological measurements | No difference in adiposity, diet, or physical activity levels in children at any timeframe |
| Gerards et al. (45) | 2015 | Netherlands | RCT | Pre-adolescents and parents | Obesity | Body composition | Children’s dietary behaviour and physical activity level, parenting practices, parental feeding style, parenting style, and parental self-efficacy | Some positive effects on parent reported child behaviours and parenting measures. No effects on children’s body composition or objectively measured physical activity |
| Eisenmann et al. (46) | 2009 | USA | RCT | Pre-adolescents and parents | Obesity | Increase physical activity, reduce screen time, and increase the consumption of fruits and vegetables | Reduction of overweight and to increase community awareness about obesity and desired behaviour changes | Small-to-modest effects for promoting children's fruit and vegetable consumption and minimizing screen time |
| Xu et al. (42) | 2015 | China | RCT | Pre-adolescents and parents | Obesity | Body composition | Behaviour and behavioural determinants | Practical and effective in improving health behaviours and obesity-related knowledge |
| Kipping et al. (48) | 2014 | England | RCT | Pre-adolescents and parents | Obesity | Physical activity and sedentary behaviour, and daily consumption of fruit and vegetables | Improve other aspects of healthy activity and diet | Not effective at increasing levels of physical activity, decreasing sedentary behaviour, and increasing fruit and vegetable consumption |
| Grydeland et al. (47) | 2013 | Norway | RCT | Pre-adolescents and parents | Obesity | BMI | Anthropometry influenced by gender, pubertal status or level of parental education | Beneficial effect on BMI and  BMI in adolescent girls, but not in boys. Children of higher educated parents seemed to benefit more |
| Villarruel et al. (50) | 2008 | Mexico | RCT | Adolescent and parents | HIV | Parent–adolescent communication, parent–adolescent sexual risk communication, and comfort with communication | None | Showed efficacy to increase the quality and quantity of parent–adolescent communication related to general and sex–specific communication |
| Jemmott III et al. (24) | 2015 | South Africa | RCT | Adolescent and parents | HIV | Unprotected vaginal intercourse | Other sexual behaviours | Significant effects |
| Miller et al. (57) | 2011 | USA | RCT | Pre-adolescent and parents | HIV | Parental perceptions of child readiness to learn about sex, HIV prevention communication, and communication effectiveness | None | Significant effects |
| Chen et al. (51) | 2009 | Bahamas | RCT | Pre-Adolescent, adolescent and parents | HIV | Sexual behaviour progression and condom use | None | FOYC effectively delays sexual risk among pre-adolescents |
| Bogart et al. (22) | 2013 | South Africa | RCT | Pre-Adolescent, adolescent and parents | HIV | Parents’ comfort in talking about sex | Parents’ self-efficacy for condom use | Significantly increased parents’ comfort with talking to their adolescent about sex |

Table 1 shows details of the 11 papers that met the inclusion criteria. All but two were conducted in the global north. Child obesity prevention interventions commonly targeted three outcomes: physical activity behaviour, dietary behaviour, and positive parenting. Primary and secondary outcomes of the six obesity studies focused on change in BMI or body composition; increasing physical activity and limiting sedentary behaviour; changing dietary behaviour; or combinations. Positive results included improving health behaviours and increased knowledge. Most studies showed no change in BMI and body composition.

The five HIV-focused interventions included target populations of pre-adolescents and/or adolescents and parents. Primary and secondary outcomes included a change in parent-adolescent communication, parent-adolescent sexual risk communication, and HIV prevention communication. Most studies showed significant effects of increased parent-learner communication.

**Table 2: Summary of interventions**

| **Classification scheme** | **Name of intervention** | **Intervention components** | **Theory based** | **Intensity** |
| --- | --- | --- | --- | --- |
| Adab et al. (49) | WAVES | The intervention encouraged healthy eating and physical activity, including a daily additional 30 minute school time physical activity opportunity, a six week interactive skill based programme in conjunction with Aston Villa football club, signposting of local family physical activity opportunities through mail-outs every six months, and termly school led family workshops on healthy cooking skills. | Not specified but recommendations for behavioural economics theory such as nudge theory | Components overlapped over a 12-month programme run throughout one school year. |
| Gerards et al. (45) | Lifestyle Triple P | The intervention strategy consisted of active skills training methods comprising parental group sessions and individual telephone sessions. Parents were instructed on a range of nutrition, physical activity and positive parenting strategies. Individual telephone sessions provided parents individual support in implementing the strategies at home. The intervention materials consisted of a parent workbook, a recipe book, and an active games booklet. | Self-regulation principles | A 14-week intervention comprising ten 90-minute parental group sessions and four individual 15–30 minute telephone sessions. |
| Eisenmann et al. (46) | Switch | The program promoted healthy active lifestyles by encouraging students to 'Switch what you Do, Chew, and View'. The community component was designed to promote awareness of the importance of healthy lifestyles and the prevention of childhood obesity in the targeted communities, and included paid advertising and unpaid media emphasizing the key messages. | Socio-ecological framework such as behavioural and environmental strategies, Brofenbrenner's ecological model, behavioural economics theory | A four month program where goals were to be active for 60 minutes or more per day, limit total screen time to two hours or less per day, and to eat five fruits/vegetables or  more per day. |
| Xu et al. (42) | CLICK-Obesity | Program had health and physical education components: classroom curriculum, (including physical education and healthy diet education); school environment support; family involvement; and fun programs/events. The classroom curriculum was designed to disseminate knowledge and skills to promote individual healthy eating behaviours and to increase inside-/outside-school physical activity. Posters were used for the school environment support. The family component provided parents (and children) with materials and resources to facilitate the adoption of the healthy target behaviours. Monthly packets containing behavioural tools were provided to assist parents and children in modifying their behaviours. The family involvement component included parents health class at school, in which they were invited to participate in an educational program twice per semester to learn appropriate strategies to advance healthy lifestyle choices against obesity. The fun programs included three competitions (picture painting, short paper writing and stage drama) were held in two semesters, no unhealthy snack week, no TV week, no soft drink week. | The theory of triadic influence and the comprehensive school health program model | One 30-minute lesson was delivered each month for one academic year (8 months). |
| Kipping et al. (48) | Active for Life Year 5 | The intervention consisted of teacher training, provision of 16 lessons and 10 child-parent interactive homework plans, all materials required for lessons and homework, and written materials for school newsletters and parents. | Social cognitive theory and self-efficacy to make behavioural change | 16 lesson plans delivered over two out of the three school terms in 6-7 months. |
| Grydeland et al. (47) | HEIA | Promotes healthy diet and to increase awareness of healthy choices, to increase participants’ physical activity during school hours and leisure time, and to reduce screen-time. Lessons with student booklet, posters for classrooms, sports equipment for recess activities, and active commuting campaigns were used for students. Fact sheets were used for parents. | Socio-ecological framework | 20 month intervention |
| Villarruel et al. (50) | None | The intervention included components for parents and adolescents including small-group discussions, videos, interactive exercises such as role– plays, and skills-building activities. The intervention also addressed the importance of family in supporting health and emphasized the principal role of parents in promoting the health of their adolescents. The HIV risk reduction intervention for parents focused on parent–adolescent communication. Parents received content about pregnancy and HIV prevention similar to that provided to their adolescents. Parents also received content to support sexual– specific communication (e.g., parental values and standards about sex, how to avoid risky situations, dealing with discomfort about communication) and parent–adolescent communication in general (e.g., aspirations for their children, creating opportunities for communication). Parents were provided with “homework” that was to be completed with their adolescent in between sessions as a means of practicing some of the communication strategies presented in the program. | Theory of reasoned action and planned behaviour | Intervention consisted of six 60–minute modules implemented on two consecutive Saturdays |
| Jemmott III et al. (24) | None | Interactive exercises, games, brainstorming, role-playing, group discussions, and comic workbooks to address issues. | Social cognitive theory and the theory of planned behaviour | Intervention consisted of 12 1-hour modules, with 2 modules delivered during each of 6 sessions on 6 consecutive school days. |
| Miller et al. (57) | Parents Matter! | Raising parents’ awareness of adolescent sexual risk behaviour and enhancing parenting skills. Intervention used discussion, videotapes, modelling, role-playing, group exercises, and homework assignments. | Social and behavioural theories. No further description. | Each of the five session lasted two and a half hours. |
| Chen et al. (51) | Focus on Youth in the Caribbean | Includes discussions, risk avoidance strategies, communication and negotiation strategies and skills, HIV-related knowledge, and condom-use skills as well as games and exercises to reinforce main messages, and a fictional family story to contextualize decision-making. | Social cognitive theory, protection motivation theory | Ten weekly session requiring about 75 minutes to complete. |
| Bogart et al. (22) | Lets Talk! | Worksite-based parenting program that improves parent-child communication about HIV and sexual health, and parent condom use self-efficacy and behaviour. | None described | Five weekly two-hour group sessions |

Table 2 provides a summarised description of the interventions. The modalities through which each intervention was performed varied. Examples of modalities / approaches targeting obesity included an interactive skill-based programme with a football club, signposting and mail-outs, workshops on healthy cooking skills, positive parenting strategies, parent workbooks, recipe books, and active games booklets. HIV-related knowledge was disseminated through interactive discussions, stories, games and exercises to reinforce messages and to contextualise decision-making.

Social or behavioural theories informed many, but not all, of the interventions. Social cognitive theory (40) provided a basis for many of the school-based intervention programmes, particularly the constructs of self-efficacy (an individual’s confidence in his/her ability to perform the desired behaviour) and behavioural capability (knowledge and skills to influence behaviour). Other theories such as triadic influence, reasoned action, and protection motivation, were also used. Other interventions drew on the socio-ecological model which acknowledged that, while behaviour in children is partly determined by themselves, they are highly dependent on both direct intervention by parents (e.g. the food provided, opportunities for physical activity) and patterns of behaviour within the family, school and peer groups (41). As children get older, the relative importance of self-directed as opposed to family-directed behaviour increases, which is then influenced by broader social factors such as the school environment and peers (41).

The needs assessment

Of the 51 eligible educators (i.e. management and Grade 6 educators) at the five schools, 46 (90.2%) consented to participate in the needs assessment survey. The participants’ demographic profile is summarised in Table 3.

**Table 3: Demographic characteristics of participants**

| **Characteristics** | **n** | **%** |
| --- | --- | --- |
| Sex  Female  Male | 33  13 | 71.7  28.3 |
| Position  Educator  Manager | 27  19 | 58.8  41.3 |
| Education  Diploma  Undergraduate degree  Postgraduate degree | 13  18  15 | 28.3  39.1  32.6 |
| Location of school  Rural  Urban | 24  22 | 52.2  47.8 |
| School classification  Quintile 1  Quintile 2  Quintile 3  Quintile 4 | 17  7  15  7 | 37.0  15.2  32.6  15.2 |

Most participants were female (71.7%); the mean age was 44.8 years, and the average teaching tenure was 16.5 years. All respondents had a post high school qualification, with 71.7% having either an undergraduate or postgraduate degree. Overall, 41.3% of all respondents were in management positions.

On average, there were nine participants per school (range 7-15), representing four quintiles: 37.0% from a quintile 1, rural school; 15.2% from a quintile 2, rural school; 32.6% from a quintile 3, urban school, and 15.2% from a quintile 4, urban school. When aggregating into rural/urban classification, 52.2% of respondents were from rural schools and 47.8% from urban schools.

Table 4 provides a summary of educators’ understanding of the relationship between health and lifestyle, and learning, and the perceived factors that influence this in their schools and communities. Most respondents (89.1%) felt that the health and lifestyle of a child has a great or very great effect on his/her learning in the classroom. Most respondents (78.3%) also identified a great or very great need for health programmes at their schools.

**Table 4: Summary of the health status of learners as perceived by educators**

| **Questionnaire item** | **Response** | **n** | **%** |
| --- | --- | --- | --- |
| 1. Perceived disease-related problems in schools | HIV  Malnutrition  Obesity  Asthma  Influenza  Tuberculosis  Diabetes  Epilepsy  Learning disability | 23  15  5  5  3  2  2  1  1 | 50.0  32.6  10.9  10.9  6.5  4.3  4.3  2.2  2.2 |
| 2. Perceived prevalence of disease in schools | To a great or very great extent  To a moderate extent  To a slight extent  Not at all | 12  18  15  1 | 26.1  39.1  32.6  2.2 |
| 3. Perceived prevalence of obesity among learners | To a great or very great extent  To a moderate extent  To a slight extent  Not at all | 4  15  21  6 | 8.7  32.6  45.7  13.0 |
| 4. Perceived levels of physical activity among learners in schools | To a great or very great extent  To a moderate extent  To a slight extent  Not at all | 20  19  7  0 | 43.4  41.3  15.2  0 |
| 5. Perceived high-risk behaviour among learners in schools | To a great or very great extent  To a moderate extent  To a slight extent  Not at all | 11  9  17  9 | 23.9  19.6  37.0  19.6 |
| 6. The perceived need for health programmes in schools | To a great or very great extent  To a moderate extent  To a slight extent  Not at all | 36  8  1  1 | 78.3  17.4  2.2  2.2 |
| 7. Perception of whether health and lifestyle affect learning | To a great or very great extent  To a moderate extent  To a slight extent  Not at all | 41  4  1  0 | 89.1  8.7  2.2  0 |

Overall, 26.1% of respondents perceived that there was a high or very high prevalence of disease among learners at their schools; and 39.1% perceived a moderate prevalence. Disease-related problems differed between rural and urban schools and between quintiles. Fewer disease-related problems were reported in urban schools (34.4%) compared to rural schools (65.6%). Asthma, obesity and influenza were the most common diseases in the urban schools; and HIV, malnutrition, obesity and TB were most common in the rural schools. Overall, 78.3% of respondents reported that obesity was a slight or moderate problem in their schools. Malnutrition was both perceived and observed to be a more significant issue at the rural schools, while obesity was perceived to be a more prominent problem in urban schools.

Logic model of the problem

The formative research facilitated the conceptualisation of the logic model of the problem (Figure 1). Specific programme objectives and outcomes were then identified.

**Figure 1: Logic model of the HIV and obesity problem**

**Step 2: Formulation of outcomes, performance objectives and change objectives**

The logic model of the HIV and obesity problem, developed in step 1, led to the identification of three key targetable behaviours: 1) modifying diet, 2) increasing physical activity, and 3) increasing learner-parent communication. These were translated into three interventions: 1) knowledge dissemination to both learner and parent, 2) translation of knowledge into change skills, and 3) strengthening autonomous support and self-efficacy in communication of learner and parents.

Performance objectives were identified to specify the action required by the target groups to modify behavioural change. The main determinants selected for the intervention design were knowledge, change skills and self-efficacy in communication.

Specific behaviours were identified as necessary to modify the determinants, which led to the identification of environmental determinants: 1) social support, 2) social norms, 3) access to resources, and 4) organisational support.

The determinants and performance objectives were then matched to develop matrices of change objectives (Table 5) for learner and parents:

- To increase health knowledge to prevent obesity and HIV infection in the short and long-term,
- To increase application of change skills to prevent obesity/underweight and HIV infection in the short and long-term, and
- To strengthen autonomous support and self-efficacy in communication of learner and parent.

**Table 5: Matrices of change objectives**

| **Change Objective I:**  Increase in health knowledge to prevent obesity and HIV infection in the short and long-term | | |
| --- | --- | --- |
| **Behavioural Outcome I:**  Behavioural intention to change through application of knowledge to avoid risky behaviour that could result in obesity and HIV infection in the short and long-term | | |
| **Performance Objective 1**:  Communicate and reinforce obesity prevention knowledge to learners and their parents | **Personal Determinant** | |
|  | **Knowledge** | Comprehend knowledge about obesity/underweight and show awareness of the advantages of proper nutrition and physical activity |
|  | **Belief** | Believe that proper nutrition and physical activity are important for overall well-being |
|  | **Attitudes** | Appreciate and value the benefits of proper nutrition and physical activity |
|  | **Self-efficacy** | Express confidence to communicate with family about strategies to support obesity prevention |
|  | **External Determinant** | |
|  | **Social Support** | Receives social reinforcement from family, educators and peers about obesity/underweight and the advantages of proper nutrition and physical activity |
|  | **Social Norms** | Appropriateness and acceptability of disseminated knowledge  Perception that peers are communicating with families and seek support from family  Modelling by parents and peers |
|  | **Access to Resources** | Availability and accessibility to nutritious foods in the home and school environment |
|  | **Organisational support** | School tuck-shops and feeding schemes to support healthy eating practices |
|  | | |
| **Performance Objective 2**:  Communicate and reinforce HIV prevention knowledge to learner and parent/s. | **Personal Determinant** | |
|  | **Knowledge** | Comprehend knowledge about HIV and show awareness of positive (sexual abstinence) and negative influences (alcohol, drugs) that could affect behaviour resulting in HIV infection in pre-adolescence and adulthood |
|  | **Belief** | Believe that positive (sexual abstinence) and negative influences (alcohol, drugs) could affect behaviour resulting in HIV infection in pre-adolescence and adulthood |
|  | **Attitudes** | Appreciate and value the benefits of positive (sexual abstinence) and negative influences (alcohol, drugs) could affect behaviour resulting in HIV infection in pre-adolescence and adulthood |
|  | **Self-efficacy** | Express confidence to communicate with family about strategies to support obesity prevention |
|  | **External Determinant** | |
|  | **Social Support** | Receives social reinforcement from family, educators and peers about HIV prevention |
|  | **Social Norms** | Appropriateness and acceptability of disseminated knowledge  Perception that peers are communicating with families and seek support from family  Modelling by parents and peers |
|  | **Access to Resources** | Availability and accessibility of health facilities to test and administer medication if necessary close to home and school environments |
|  | | |
| **Change Objective II**  Application of change skills to prevent obesity/underweight and HIV infection in the short and long-term | | |
| **Behavioural Outcome II**  Behavioural intention to change by application of skills to prevent obesity/underweight and HIV infection in the short and long-term | | |
| **Performance Objective 3**:  Encourage additional learning from activity-based change skills for obesity prevention | **Personal Determinant** | |
|  | **Knowledge** | Comprehend knowledge skills about obesity/underweight and show awareness of the advantages of proper nutrition and physical activity |
|  | **Change Skills** | Demonstrates skills to family and seek/offer support |
|  | **Belief** | Believe that proper nutrition and physical activity are important for overall well-being |
|  | **Attitudes** | Appreciate and value the benefits of proper nutrition and physical activity |
|  | **Self-efficacy** | Express confidence to communicate with family about strategies to support obesity prevention |
|  | **External Determinant** | |
|  | **Social Support** | Encouragement from family, educators and peers to perform activity and to learn the skill set |
|  | **Social Norms** | Appropriateness and acceptability of activity or skill set to be learnt and applied  Modelling by family and peers |
|  | | |
| **Performance Objective 4**:  Encourage additional learning from activity-based change skills for HIV prevention | **Personal Determinant** | |
|  | **Knowledge** | Comprehend knowledge skills about obesity/underweight and show awareness of the disadvantages of risky behaviour |
|  | **Change Skills** | Demonstrates skills to family and seek/offer support |
|  | **Belief** | Believe that proper communication is important for overall support and well-being |
|  | **Attitudes** | Appreciate and value the benefits of not indulging in risky behaviour |
|  | **Self-efficacy** | Express confidence to communicate with family about strategies to support HIV prevention |
|  | **External Determinant** | |
|  | **Social Support** | Encouragement from family, educators and peers to perform activity and to learn the skill set |
|  | **Social Norms** | Appropriateness and acceptability of activity or skill set to be learnt and applied  Modelling by family and peers |
|  | | |
| **Change Objective III**  Strengthen autonomous support and self-efficacy of learner and parent through dissemination of knowledge and change skills for improved communication | | |
| **Behavioural Outcome III**  Improved learner-parent communication to avoid risky behaviour that may result in HIV infection | | |
| **Performance Objective 5**:  Encourage learner-parent communication about HIV prevention in open informal discussion during family time | **Personal Determinant** | |
|  | **Knowledge** | Comprehend knowledge about how to communicate with family  Comprehend knowledge about risky behaviour and its linkage to HIV |
|  | **Change Skills** | Demonstrate skills to communicate with family and seek/offer support |
|  | **Belief** | Believe that communication and discussion of risky behaviour and its linkage to HIV are important to prevent negative consequences |
|  | **Attitudes** | Appreciate and value the benefits of communication and discussion of risky behaviour and its linkage to HIV |
|  | **Self-efficacy** | Confidence to communicate and converse with family about difficult topics such as HIV prevention and strategies to support prevention |
|  | **External Determinant** | |
|  | **Social Support** | Receives social reinforcement from family, educators and peers |
|  | **Social Norms** | Perception that peers are communicating with families and seek support from family |

**Step 3: Selection of theory-based methods and practical strategies**

The identification and selection of theoretical constructs required to influence change among the selected determinants were based on theoretical methods detailed by Kok et al. (2015) (42). Practical strategies were identified to apply the theoretical methods to practice. These delivery strategies, aimed at changing determinants, were organised into a coherent programme of practical strategies so that relevant methodology supplemented the logic model of change. The strategies were required to fit the implementation context; be applicable, and be acceptable to the learner and their parents. The theory-based methods were translated into practical strategies that were matched to the change objectives from the matrices.

**Table 6: Practical strategies for intervention design**

| **Change Objective I**  Increase in health knowledge to prevent obesity and HIV infection in the short and long-term | |
| --- | --- |
| **Performance Objective 1** | Communicate and reinforce obesity prevention knowledge to learner and their parents |
| **Determinant** | Knowledge |
| **Theoretical Method** | Advanced Organisers  (Theories of information processing) |
| **Implementation Strategy** | Planned educator-delivered lessons at school for learner with notes that parents will also read through. Parents are expected to sign after reading through each lesson.  The learner will be asked to verbally convey messages learnt during lesson time to their parents at home for reinforcement.  Games and puzzles for reinforcement. |
|  | |
| **Performance Objective 2** | Communicate and reinforce HIV prevention knowledge to learner and parents |
| **Determinant** | Knowledge |
| **Theoretical Method** | Advanced Organisers  (Theories of information processing) |
| **Implementation Strategy** | Planned educator-delivered lessons at school for learner with notes that parents will also read through. Parents are expected to sign after reading through each lesson.  The learner will be asked to verbally convey messages learnt during lesson time to their parents at home for reinforcement.  Games and puzzles for reinforcement. |
|  | |
| **Change Objective II**  Increase in knowledge and application of change skills to prevent obesity/underweight and HIV infection in the short and long-term | |
| **Performance Objective 3** | Encourage additional learning from activity-based change skills for obesity prevention |
| **Determinant** | Change skills |
| **Theoretical Method** | Guided Practice  (Social Cognitive Theory and Theories of Self-Regulation) |
| **Implementation Strategy** | Planned educator-delivered lessons of guided practice of skill at school for learner, with notes that parents will also read through. Parents are expected to sign after reading through each lesson.  The learner will be asked to verbally convey skills learnt and practised during lesson time to their parents at home for reinforcement.  There will be an activity for parent and learner to perform together as parent assisted homework. |
| **Performance Objective 4** | Encourage additional learning from activity-based change skills for HIV prevention |
| **Determinant** | Change skills |
| **Theoretical Method** | Guided Practice  (Social Cognitive Theory and Theories of Self-Regulation) |
| **Implementation Strategy** | Planned educator-delivered lessons of guided practice of skill at school for learner, with notes that parents will also read through. Parents are expected to sign after reading through each lesson.  The learner will be asked to verbally convey skills learnt and practised during lesson time to their parents at home for reinforcement.  There will be an activity for parent and learner to perform together as parent assisted homework. |
|  | |
| **Change Objective III**  Strengthen autonomous support and self-efficacy of learner and parent through dissemination of knowledge and change skills for improved communication | |
| **Performance Objective 5** | Encourage learner-parent communication about HIV prevention in open, informal discussion during family time |
| **Determinant** | Self-efficacy |
| **Theoretical Method** | Discussion (Theories of Information Processing) |
| **Implementation Strategy** | HIV Communication Exercise for parent and learner to converse about HIV issues in an informal discussion. |
|  | |

**Step 4: Intervention Development**

Health promotion (the provision of information, education and life skills for improved health outcomes) assists in making a healthy choice an easy choice, by creating a supportive environment, and enables people to have more control over their health. Five priority areas of action for health promotion recommended by the Ottawa Charter are: re-orientation of health care services toward illness prevention and health promotion; development of personal skills; strengthening of community action; creation of supportive environments; and building of healthy public policy (43). The Charters’ action areas, together with theory- and EBP, and collaboration with key stakeholders, played an instrumental role in the development of the intervention (43).

As a result of the formative research, the intervention design was based on the field intervention framework from the CLICK-Obesity Programme (44,45). Strategies targeting different components were also selected from other EBPs to address and achieve the desired behavioural outcomes in the context of the study (Table 7). Some selected components were incorporated unchanged while others were adapted to suit the context of the study.

Adaptation is defined as the process of modifying key characteristics of an intervention, recommended activities and delivery methods, without competing with or contradicting the core elements, theory and internal logic of the intervention thought most likely to produce the main effects (46). Certain EBP components were tailored for several reasons. Firstly, this intervention included the integration of both NCD and CD, and did not address a particular disease as in other studies. Secondly, the content, duration, and dose of lessons delivered differed, due to the time-constraints of implementing during lesson time and the alignment with the existing Grade 6 curriculum. Thirdly, it was based on educator preference for content and design to suit the culture of the community and be age-appropriate for pre-adolescents. Lastly, details of some EBP components were not ideally documented.


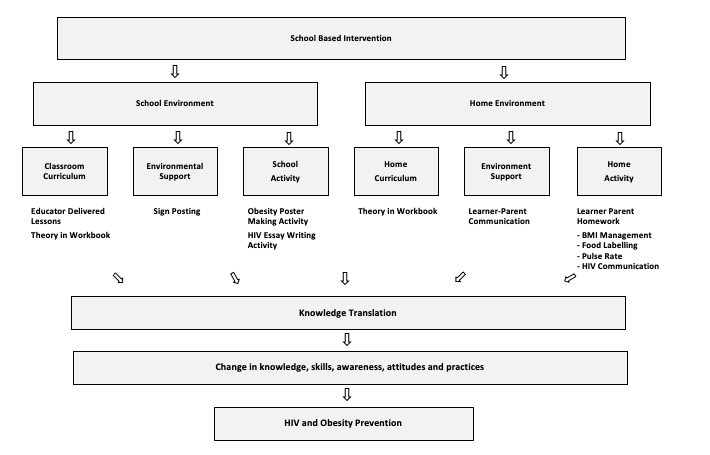


**Figure 2: The CIrCLE of Life Initiative**

The CIrCLE of Life Initiative

The design of The CIrCLE (Child Influencing paRent Communication for Life Education) of Life Initiative (Figure 2) has both a school and home environmental component, each of which comprises a learning curriculum, environmental support, and activity-based constituents. The school component targets the learner while the home environment component targets the parent.

***The School Environment***

*Classroom Curriculum:* The classroom curriculum for learners require delivery of 10 30-minute lessons delivered by educators within a classroom setting. Educators will be provided with training and resources for lesson delivery. Table 7 specifies the lesson plan.

**Table 7: Outline lesson plans in curriculum**

| **Lesson** | **Topic** | **Source adaptation** |
| --- | --- | --- |
| **Lesson 1**  **Lesson 2** | - Understanding obesity, underweight and normal weight - Healthy body image - Measuring and interpreting body mass index | Gerards et al. (45), Eisenmann et al. (46), Xu et al. (42), Grydeland et al. (47), Kipping et al. (48) |
| **Lesson 3**    **Lesson 4** | - The five food groups - Health benefits of the food groups - Social marketing - Sugar and salt intake - Interpreting food labels | Adab et al. (49), Gerards et al. (45), Eisenmann et al. (46), Xu et al. (42), Grydeland et al. (47), Kipping et al. (48) |
| **Lesson 5**  **Lesson 6** | - Understanding Physical Activity - Limiting sedentary activity - Measuring and interpreting pulse rate | Gerards et al. (45), Eisenmann et al. (46), Xu et al. (42), Grydeland et al. (47), Kipping et al. (48) |
| **Lesson 7**  **Lesson 8** | - Understanding HIV/AIDS - Avoiding risky situations - Making sexual decisions and understanding your values - Personalising the risks | Villarruel et al. (50), Dinaj-Koki et al. (27), Jemmott III et al. (24), Miller et al. (23), Chen et al. (51), Bogart et al. (22) |
| **Lesson 9**  **Lesson 10** | - Parent-adolescent communication in general - Support for sexual-specific communication - Dealing with discomfort about communication - Assertive communication skills - Learner-parent communication exercise | Villarruel et al. (50), Dinaj-Koki et al. (27), Jemmott III et al. (24), Miller et al. (23), Chen et al. (51), Bogart et al. (22) |

Grade 6 learners will be chosen as participants because the developed lessons are aligned with their existing syllabus. These learners are also in the pre-adolescent phase, and will not be lost to follow up if the intervention runs longer than scheduled. Promoted Grade 7 learners are required to change schools as they move into high school.

Although the lessons are designed to be part of the life orientation lesson, they can also be taught during other subject lessons – as part of a transdisciplinary lesson. For example, measuring BMI can be covered during the mathematics lesson to reinforce the mathematics involved, or pulse rate can be taught during the physical education lesson while exercising. This will be a choice of the implementers at each school.

*Environment Support:* The school environment can be advantageous to help learners make desirable health-related behavioural changes and maintain a healthy lifestyle. Brief health-related messages on posters, designed by learners themselves will be displayed at schools, in the playground and tuck-shop locations. The posters will be updated accordingly to scheduled intervention themes. This component was adapted from Adab et al. (47) and Xu et al. (44,45).

*School Activity:* This comprises two competitions. The first is a poster-making competition to be held after Lesson 6, and the second is an HIV essay writing competition to be held after Lesson 8. Prizes will be awarded to winners at each school. This component was adapted from Xu et al. (44,45) to fit in with the existing school curriculum.

***Home Environment***

*Home Curriculum:* Parents are the gatekeepers for their children’s food choices and serve as role models by practising and reinforcing healthy lifestyle choices. Family involvement strategies are included to introduce families to the intervention and to assist them to create a supportive environment to help children change unhealthy behaviours. Parents are to read through and sign a workbook after each lesson. Only the strategy was adapted from Gerards et al. (48) and had to be customised to suit the integrated intervention.

*Environment Support:* The learners will be asked during class lessons to communicate lesson information to their parents.

*Home Activity:* Learner-parent homework was included in a workbook to reinforce the lesson delivered at school. This strategy was adapted from Kipping et al. (49) but the content had to be tailored. Homework activities include: measuring and interpreting BMI; interpreting food labels; measuring and interpreting pulse rate; and learner-parent communication exercises.

***Resource Development***

Educator training manuals, lesson curricula, and learner-parent workbooks were developed. Educators were asked to comment on the cultural fit, acceptability, appropriateness, attractiveness, completeness, and relevance of all programme material. Educators, learners and parents pre-tested the intervention materials and their recommendations for changes were included in the tools.

The workbook included lesson notes, a description of the skill, and reinforcement activities. The parent will be required to sign at the end of each lesson. A sticker reward system was developed to encourage learner-parent participation. If all stickers in the workbook are collected, the learner will be awarded a prize at the end of implementation (a game that reinforced positive habits).

**Step 5: Adoption and Implementation**

Stakeholder engagement and collaboration ensured refinement of the intervention. Various key stakeholders included pre-adolescent learners and their parents, educators, representatives from the sectors of health and education, and non-Governmental organisations. Intervention concepts were explored informally with various stakeholders that influenced the adoption of the intervention by tailoring the intervention to fit local needs, available resources and competencies.

Educators played an advisory role regarding the timing of programme implementation, selection of suitable teaching techniques, and programme materials. Educators’ suggestions included increasing parental involvement by using incentives; group work to deal with the problem of too many learners per class; and incorporating fun and enrichment exercises into the lessons by using games and activities. The representatives from the Departments of Health and Education played a key role in ensuring that the objectives, methods and strategies were in line with the education curriculum. Consultation with non-governmental organisations and health specialists (nutritionist, exercise therapist, occupational therapist, physiotherapist and child psychologist) also informed the intervention.

**Step 6: Evaluation Plan**

Process and outcome evaluation strategies for implementation of the intervention were developed. The variables of implementation fidelity, acceptability, appropriateness, feasibility, sustainability, cost, adoption and penetration will be assessed during the process evaluation, based on two evaluation tools that were developed during this step. The first tool, an observation form, will be completed by the researcher based on observations made during lessons. The second tool, an in-depth questionnaire booklet, will be completed by the educator after each delivered lesson.

The outcome evaluation will assess if the learning objectives were achieved and whether the envisaged determinants of the health-promoting behavioural intention to change were adopted. Learner and parent pre- and post-implementation questionnaire tools were developed to measure the change in knowledge, skills, behavioural intention to change, attitudes, beliefs and self-efficacy in communication.

**Discussion**

The unique characteristics of CDs and NCDs posed a challenge in proposing an integrated solution to address the double burden of disease. However, features common to both categories of disease assisted in the integration of various health promotion and prevention initiatives into a combined intervention. Although the inclusion of EBP was vital to the overall success, only selected components from individual interventions could be incorporated, due to the diversity of the diseases.

The IM approach (33) facilitated the development process and informed the content of *The CIrCLE of Life Initiative.* Although IM is typically used in the development of innovative health interventions, the versatility of the protocol which could be adapted as an approach to describe the adaptation, adoption, implementation and sustainability of a combination of EBPs, was valuable. The IM tool was also beneficial in screening EBPs and customising the interventions to the specific target population. To maximise the effectiveness of the intervention and increase the likelihood of long-term programme sustainability, the core elements of the selected EBP parameters were maintained. The challenge, however, was that specific material and in-depth programme content were sometimes limited or unavailable in the published literature, necessitating the restructuring of certain intervention elements.

A further challenge of adapting EBPs was identifying and assessing factors that impede replicability. Social determinants of behaviours may be influenced by the home environment or socio-economic challenges, which may affect intervention outcomes. Behaviour change is especially difficult when resources are unavailable, or circumstances do not allow for this (e.g. affordability of fresh fruit and vegetables to maintain nutritional diets). EBP components that proved effective in other environments may not necessarily work in the selected study context, warranting a need for strategies to be thoroughly thought through.

The scoping review revealed that some EBPs had shown poor outcomes in attempting to alter anthropometry in the school environment (47,48); however, efforts to decrease behavioural risks have had positive outcomes. The design of this intervention therefore omitted anthropometry as an outcome and focused on knowledge dissemination and skill transfer.

In the needs assessment which offered insight into the health status of the learners, HIV and malnutrition were highlighted as the most significant disease-related problems in the schools. This was contrary to the South African general population statistics that show obesity to be high among adults (10), and an increasing problem in children (10). Although obesity was prevalent, malnutrition was a greater challenge amongst learners. These findings prompted changes to the initially proposed intervention that focussed on addressing obesity, to include malnutrition and healthy nutrition.

Positioning questions included in the needs assessment not only confirmed that educators understood the causal link between health and learning in the classroom, but also gave impetus to the relevance and appropriateness of the need for a health programme and the purpose for which it was designed. This gave insight into how a transdisciplinary intervention would be received at the school, the intervention acceptance, and key stakeholder buy-in.

The logic model (Figure 1) is a graphical/textual representation of how the intervention is intended to work, and depicts what the intervention will accomplish (50). It helped make underlying assumptions about the intervention explicit, and provided a common approach to integrating planning, implementation and evaluation. The logic model for understanding the intervention objectives was linear and focused on the assumed cause-effect relationship identified in theoretical and empirical research. However, the proposed outcomes were non-linear and part of a multifaceted system, emphasising the need for a transdisciplinary approach.

*The CIrCLE of Life Initiative* is considered to be a transdisciplinary intervention for two reasons. First, it unites diverse perspectives from two disciplines, i.e. health and education. Second, in teaching health in the classroom, it unites diverse perspectives from different subject disciplines such as English, art, life orientation and mathematics. The intervention was based on integrative cross-disciplinary collaboration. Stakeholders from the Departments of Health and Education collaborated to share knowledge that extended beyond their disciplinary perspectives. The transdisciplinary approach was used to increase uptake, ensure consideration of realistic concerns and constraints, and yield sustainable outcomes. Given stakeholders’ diverse backgrounds and roles, their perspectives were instrumental in strengthening the intervention infrastructure and improving its organisational functioning.

Stakeholder engagement also ensured the refinement of the priority areas during the planning and development processes by providing parameters for effectiveness that fit with the target population, culture and context during the adaptation process. Stakeholder engagement also encouraged and created a sense of ownership during the programme development. Utilising the bottom-up approach, by including stakeholders in the planning process, increased user-perspective and the likelihood of program sustainability. Although the stakeholder consultations involved relatively few participants, they represented a broad spectrum of key stakeholders influencing the intervention design.

As a reinforcement strategy for learners, a workbook that included a range of puzzles and activities, such as colouring-in pictures, crosswords and word searches, was designed. This was based on the premise that children learn through play and activities (51). The learners will be asked to converse with their parents about daily classroom lessons and activities as well as complete parent-assisted homework. Such additional reinforcement is vital to facilitate further knowledge and skills expansion beyond the educational environment.

Poster and story-writing activities were introduced to engage learners in the development of some of the material with the intent to increase receptiveness and responsiveness to health communication. Learner assessment rubrics for these activities were also prepared for educators.

Communicating healthy lifestyle messages from learner to parent, and seeking their help and support, requires a change in personal and external determinants. As a personal determinant, the learner requires specific communication knowledge and skills to transfer the message to his/her parent. This was the reasoning behind the inclusion of a communication strategy within the intervention. As an external determinant, the learner also requires both support and reinforcement from his/her parents, educators and peers. The intervention was structured to encourage a two-way communication support process between parent and child to further reinforce health messages, with mutual benefits to both.

Parental involvement is a critical constituent in achieving the target behaviours. The intervention not only offers a focused and intensive approach by improving knowledge and skills related to health, but also addresses broader parenting skills. Parenting programmes from EBPs have shown that parenting communication skills are fundamental to health behaviour and practices (52), especially when it comes to HIV and sex-related health practices (23).

There were several strengths of the study. The IM approach, with its systematic and detailed planning, optimised the chances for intervention adoption, implementation and sustainability. It incorporated theory, evidence and guidance on how to develop the intervention, in partnership with key stakeholders, that resulted in a coherent intervention model. The approach also provided a useful foundation for the development of the intervention, particularly the goals. Furthermore, it contributed to the integrity of the study and the development of the instruments.

The IM provided a useful checklist where each step had a clear purpose and place in the intervention; however, one of the limitations was its lengthy processes. The time and effort taken to complete each step, and the complexity that it entailed, were arduous. This experience is similar to the findings from other research (26,53,54).

The IM process was cumbersome when considering complex behaviours and working with different disease components, i.e. NCDs and CDs. As a result, many behaviour change techniques that were generated were not necessarily coherent or compatible. It became necessary to target only critical behaviours so that the intervention strategy was focused and related to the key programme objectives.

**Conclusion**

The development of an integrated CD (HIV) and NCD (obesity) transdisciplinary intervention was grounded on proficiency from both theoretical- and EBP. Theoretical practices, such as the taxonomies of behaviour change, amalgamated with the application of the IM heuristic, were assimilated into the design and development of *The CIrCLE of Life Initiative*. Adaption of EBPs ensured that the intervention fit the target population, culture and context, and increased the chance of sustainability. Stakeholder engagement and collaboration supported the process, with the provision of beneficial parameters that resulted in the preparation of knowledge and skill-enhancing practical strategies for dissemination, adoption, implementation and sustainability.

**Abbreviations**

AIDS: Acquired Immunodeficiency Syndrome; BMI: Body Mass Index; CD: Communicable Disease; CIrCLE: Child Influencing paRent Communication for Life Education*;* EBP: Evidence-Based Practice; HIV: Human Immunodeficiency Virus; IM: Intervention Mapping; NCD: Non-Communicable Disease; PRISMA: Preferred Reporting Items for Systematic reviews and Meta-Analyses PRISMA-ScR: Preferred Reporting Items for Systematic reviews and Meta-Analyses extension for Scoping Reviews RCT: Randomised Control Trial; TB: Tuberculosis; TIDieR: Template for Intervention Description and Replication.

**Declarations**

**Acknowledgements**

We would like to thank all the members of the intervention development group and organisations for their participation.

**Author's contributions**

KA conceived the idea for the study and designed the study. KA drafted the manuscript with the help of NC and GN. Both NC and GN critically revised the manuscript. All authors have given their final approval and agree to be accountable for all aspects of the work in ensuring that questions related to the accuracy or integrity of any part of the work are appropriately investigated and resolved. All authors read and approved the final manuscript.

**Availability of data and materials**

The datasets and materials used and/or analysed during the current study are available from the corresponding author on reasonable request.

**Competing interests**

The authors declare that they have no competing interests.

**Consent for publication**

Written informed consent was obtained from the participants for publication of their individual details. The consent form is held by the authors and is available for review by the Editor-in-Chief.

**Ethics approval and consent to participate**

Approval to conduct the study was gained from the Human Research Ethics Committee of the Health Sciences Faculty, University of the Witwatersrand.

**Funding**

This publication is independent research funded by a postgraduate training scholarship from the Fogarty and NIAID, the UNC-Wits AIDS Implementation Science and Cohort Analyses Training Grant (Grant number 5D43TW009774-02). The views expressed in this publication are those of the authors.

**Author details**

^1^School of Public Health, University of the Witwatersrand, 27 St Andrews Road, Parktown, Johannesburg,

2193, South Africa.
^2^ Nicola.Christofides@wits.ac.za

^3^ Gill.Nelson@wits.ac.za

**References**

1. WHO. Mortality 2015 and 2030 - Baseline Scenario WHO Regions [Internet]. Projections of mortality and causes of death, 2015 and 2030. 2012 [cited 2017 Jun 14]. Available from: http://www.who.int/healthinfo/global_burden_disease/projections/en/

2. Temu F, Leonhardt M, Carter J, Thiam S. Integration of non-communicable diseases in health care: Tackling the double burden of disease in African settings. Pan Afr Med J. 2014;18:1–6.

3. Centre for Disease Control and Prevention. The Social-Ecological Model : A Framework for Prevention [Internet]. 2018 [cited 2018 Dec 1]. Available from: https://www.cdc.gov/violenceprevention/overview/social-ecologicalmodel.html

4. Stuckler D, Siegel K. Sick Societies: Responding to the global challenge of chronic disease. Oxford University Press; 2011.

5. Naidoo K, Gengiah S, Yende-Zuma N, Padayatchi N, Barker P, Nunn A, et al. Addressing challenges in scaling up TB and HIV treatment integration in rural primary healthcare clinics in South Africa (SUTHI): A cluster randomized controlled trial protocol. Implement Sci. 2017;12(1):1–12.

6. Kruger HS, Puoane T, Senekal M, van der Merwe M-T. Obesity in South Africa: challenges for government and health professionals. Public Health Nutr. 2005;8(05).

7. Mayosi BM, Flisher AJ, Lalloo UG, Sitas F, Tollman SM, Bradshaw D. The burden of non-communicable diseases in South Africa. Lancet [Internet]. 2009;374(9693):934–47. Available from: http://dx.doi.org/10.1016/S0140-6736(09)61087-4

8. Puoane T, Steyn K, Bradshaw D, Laubscher R, Fourie J, Lambert V, et al. Obesity in South Africa: The South African Demographic and Health Survey. Obes Res. 2002;10(10):1038–1048.

9. World Health Organization (WHO). WHO:Europe | Nutrition - Body mass index - BMI [Internet]. [cited 2019 Feb 22]. Available from: http://www.euro.who.int/en/health-topics/disease-prevention/nutrition/a-healthy-lifestyle/body-mass-index-bmi

10. Statistics South Africa. South African Demographic and Health Survey 2016: Key Indicator Report. Statistics South Africa. 2016.

11. Rossouw H, Grant C, Viljoen M. Overweight and obesity in children and adolescents: The South African problem. S Afr J Sci. 2012;108:1–7.

12. Sahoo K, Sahoo B, Choudhury AK, Sofi NY, Kumar R, Bhadoria AS. Childhood obesity: causes and consequences. J Fam Med Prim care [Internet]. 2015;4(2):187–92. Available from: http://www.ncbi.nlm.nih.gov/pubmed/25949965%5Cnhttp://www.pubmedcentral.nih.gov/articlerender.fcgi?artid=PMC4408699

13. Xu S, Xue Y. Pediatric obesity: Causes, symptoms, prevention and treatment (review) [Internet]. Experimental and Therapeutic Medicine. Spandidos Publications; 2016. Available from: https://doi.org/10.3892/etm.2015.2853

14. Wyatt SB, Winters KP, Dubbert PM. Overweight and obesity: Prevalence, consequences, and causes of a growing public health problem. Am J Med Sci. 2006;331(4):166–74.

15. Statistics South Africa. Mid-year Population Estimates [Internet]. 2017. Available from: http://www.statssa.gov.za/publications/P0302/P03022018.pdf

16. Mkumbo K, Schaalma H, Kaaya S, Leerlooijer J, Mbwambo J, Kilonzo GAD. The application of Intervention Mapping in developing and implementing school-based sexuality and HIV / AIDS education in a developing country context : The case of Tanzania. Scand J Public Health. 2009;37(October 2007):28–36.

17. Getahun H, Gunneberg C, Granich R, Nunn P. HIV Infection–Associated Tuberculosis: The Epidemiology and the Response. Clin Infect Dis. 2010;50(s3):S201–S207.

18. Lönnroth K, Jaramillo E, Williams BG, Dye C, Raviglione M. Drivers of tuberculosis epidemics: The role of risk factors and social determinants. Soc Sci Med. 2009;68(12):2240–2246.

19. Phaswana-Mafuya N, Peltzer K, Chirinda W, Musekiwa A, Kose Z. Sociodemographic predictors of multiple non-communicable disease risk factors among older adults in South Africa. Glob Health Action. 2013;6(1).

20. Balbus JM, Barouki R, Birnbaum LS, Etzel RA, Gluckman SPD, Grandjean P, et al. Early-life prevention of non-communicable diseases. Lancet. 2013;381(9860):3–4.

21. Harrison, A., Newell, L. M, Imrie, J., et al. HIV prevention for South African youth: which interventions work? A systematic review of current evidence. 2010;10.

22. Manuscript A, Bogart LM, Skinner D, Thurston IB, Yoesrie T, Klein DJ, et al. Let’s Talk!, A South African Worksite-Based HIV Prevention Parenting Program. J Adolesc Heal. 2013;31(9):1713–23.

23. Miller KS, Lasswell SM, Riley DB, Poulsen MN. Families Matter! Presexual risk prevention intervention. Am J Public Health. 2013;103(11):16–20.

24. Jemmott III JB, Jemmott LS, Ann O’Leary LF, Ngwane Z, Icard LD, Bellamy SL, et al. School-Based Randomized Controlled Trial of an HIV/STD Risk-Reduction Intervention for South African Adolescents. Arch Pediatr Adolesc Med. 2015;164(10):923–9.

25. Kessel F, Rosenfield PL. Toward Transdisciplinary Research. Historical and Contemporary Perspectives. Am J Prev Med. 2008;35(2 SUPPL.).

26. Lloyd JJ, Logan SL, Greaves CJ, Wyatt KM. Evidence, theory and context - using intervention mapping to develop a school-based intervention to prevent obesity in children. Int J Behav Nutr Phys Act [Internet]. 2011;8(73):15p-15p. Available from: http://simsrad.net.ocs.mq.edu.au/login?url=http://search.ebscohost.com/login.aspx?direct=true&db=ccm&AN=104672477&site=ehost-live

27. Dinaj-Koci V, Chen X, Deveaux L, Lunn S, Li X, Wang B, et al. Developmental Implications of HIV Prevention During Adolescence: Examination of the Long-Term Impact of HIV Prevention Interventions Delivered in Randomized Controlled Trials in Grade Six and in Grade 10. Youth Soc. 2015;47(2):151–72.

28. Baltussen R, Mikkelsen E, Tromp N, Hurtig A, Byskov J, Olsen Ø, et al. Balancing efficiency , equity and feasibility of HIV treatment in South Africa – development of programmatic guidance. Bio med Cent. 2013;1–9.

29. Zimmer-Gembeck MJ, Helfand M. Ten years of longitudinal research on U.S. adolescent sexual behavior: Developmental correlates of sexual intercourse, and the importance of age, gender and ethnic background. Dev Rev. 2008;28(2):153–224.

30. Damerell P, Howe C, Milner-Gulland EJ. Child-orientated environmental education influences adult knowledge and household behaviour. Environ Res Lett. 2013;8(1):015016.

31. Gardiner M. Priority Areas for Educational Attention in South Africa by Michael Gardiner. Johannesburg; 2006.

32. The Government Gazette. The national norms and standards for school funding. Pretoria; 2006.

33. Bartholomew KL, Parcel GS, Kok G GN. Planning Health Promotion Programs : An Intervention Mapping Approach. Second Edi. San Fransisco: Jossey-Bass; 2011.

34. Stea TH, Haugen T, Berntsen S, Guttormsen V, Øverby NC, Haraldstad K, et al. Using the Intervention Mapping protocol to develop a family-based intervention for improving lifestyle habits among overweight and obese children : study protocol for a quasi-experimental trial. BMC Public Health. 2016;16(1092).

35. Verbestel V, Henauw S De, Maes L, Haerens L, Mårild S, Eiben G, et al. Using the intervention mapping protocol to develop a community-based intervention for the prevention of childhood obesity in a multi-centre European project : the IDEFICS intervention. Int J Behav Nutr Phys Act. 2011;8(82):1–15.

36. Arksey H, O’Malley L. Scoping studies: towards a methodological framework. Int J Soc Res Methodol [Internet]. 2005;8(1):19–32. Available from: http://www.tandfonline.com/doi/abs/10.1080/1364557032000119616

37. Tricco AC, Lillie E, Zarin W, O’Brien KK, Colquhoun H, Levac D, et al. PRISMA Extension for Scoping Reviews (PRISMA-ScR): Checklist and Explanation. Ann Intern Med [Internet]. 2018;169(7):467–73. Available from: http://www.ncbi.nlm.nih.gov/pubmed/30178033

38. Liberati A, Altman DG, Tetzlaff J, Mulrow C, Gøtzsche PC, Ioannidis JPA, et al. The PRISMA statement for reporting systematic reviews and meta-analyses of studies that evaluate health care interventions: explanation and elaboration. Vol. 62, Journal of clinical epidemiology. 2009. e1-34 p.

39. Hoffmann TC, Glasziou PP, Boutron I, Milne R, Perera R, Moher D, et al. Better Reporting of Interventions: Template for Intervention Description and Replication (TIDieR) Checklist and Guide. Gesundheitswesen [Internet]. 2016;78(3):175–88. Available from: http://dx.doi.org/doi:10.1136/bmj.g1687

40. Bandura A. Social foundations of thought and action : a social cognitive theory / Albert Bandura. Englewood Cliffs, N.J: Prentice-Hall, 1986. xiii, 617 pp. 1986. 2–xiii, 617 p.

41. Lloyd JJ, Logan S, Greaves CJ, Wyatt KM. Evidence , theory and context - using intervention mapping to develop a school-based intervention to prevent obesity in children. 2011;1–15.

42. Kok G, Gottlieb NH, Peters G-JY, Mullen PD, Parcel GS, Ruiter RAC, et al. A taxonomy of behaviour change methods: an Intervention Mapping approach. Health Psychol Rev. 2015;1–16.

43. World Health Organization. The Ottawa Charter for Health Promotion. 2013.

44. Xu F, Ware RS, Leslie E, Tse LA, Wang Z, Li J, et al. Effectiveness of a randomized controlled lifestyle intervention to prevent obesity among Chinese primary school students: Click-obesity study. PLoS One. 2015;10(10):1–12.

45. Xu F, Ware RS, Tse LA, Wang Z, Hong X, Song A, et al. A school-based comprehensive lifestyle intervention among chinese kids against obesity (CLICK-Obesity): rationale, design and methodology of a randomized controlled trial in Nanjing city, China. BMC Public Health. 2012;12(1):316–23.

46. Mckleroy VS, Galbraith JS, Cummings B, Jones P, Harshbarger C, Collins C, et al. Adapting Evidence – Based Behavioral Interventions for New Settings and Target Populations. 2006;59–73.

47. Adab P, Pallan MJ, Lancashire ER, Hemming K, Frew E, Griffin T, et al. A cluster-randomised controlled trial to assess the effectiveness and cost-effectiveness of a childhood obesity prevention programme delivered through schools, targeting 6–7 year old children: the WAVES study protocol. BMC Public Health. 2015;15(1):488.

48. Gerards SMPL, Dagnelie PC, Gubbels JS, Van Buuren S, Hamers FJM, Jansen MWJ, et al. The effectiveness of lifestyle triple P in the Netherlands: A randomized controlled trial. PLoS One. 2015;10(4):1–18.

49. Kipping RR, Howe LD, Jago R, Campbell R, Wells S, Chittleborough CR, et al. Effect of intervention aimed at increasing physical activity, reducing sedentary behaviour, and increasing fruit and vegetable consumption in children: Active for Life Year 5 (AFLY5) school based cluster randomised controlled trial. BMJ. 2014;348(May):1–13.

50. W.K. Kellogg Foundation. Logic Model Development Guide. W.K. Kellogg Foundation Logic Model Development Guide. 2004.

51. Undiyaundeye F a. How Children Learn Through Play. J Emerg Trends Educ Res Policy Stud. 2013;4(3).

52. Gerards, S.M; Dagnelie, P.C; Jansen, M.W;v an der Goot, L.O; de Vries, N.K; Sanders MR. Lifestyle Triple P: a parenting intervention for childhood obesity Gerards S.M. BMC Public Health. 2012;12:267.

53. Detaille SI, Gulden JWJ Van Der, Engels JA, Heerkens YF, Dijk FJH Van. Using intervention mapping ( IM ) to develop a self-management programme for employees with a chronic disease in the Netherlands. 2010;10(353).

54. de Villiers A, Steyn NP, Draper CE, Hill J, Dalais L, Fourie J, et al. Implementation of the HealthKick intervention in primary schools in low-income settings in the Western Cape Province, South Africa: a process evaluation. BMC Public Health [Internet]. 2015;15(1):818. Available from: http://bmcpublichealth.biomedcentral.com/articles/10.1186/s12889-015-2157-8

**Figure legends**

**Fig. 1 A logic model of the HIV and obesity problem.** A detailed overview of the inputs, processes, outputs, short- and long-term outcomes required to create an impact.

**Fig. 2 The CIrCLE of Life Initiative.** The CIrCLE of Life Initiative has both a school and home environmental component, each of which comprises a learning curriculum, environmental support, and activity-based constituents. The school component targets the learner while the home environment component targets the parent. This may result in knowledge translation, a change in knowledge, skills, awareness, attitude and practices with the ultimate aim of HIV and obesity prevention.

**Additional files**

Additional file 1. The PRISMA-ScR Checklist. (DOCX 111 kb)

Additional file 2. The PRISMA Flow Diagram. (.DOC 31kb)

Additional file 3. Scoping review search strategy. (DOCX 13 kb)

Additional file 4. The TIDieR Checklist. (DOCX 16 kb)
