# Supplementary material for: Educators’ perceptions of organisational readiness for implementation of a pre-adolescent transdisciplinary school health intervention for inter-generational outcomes
Source: PLoS One. 2020 Jan 8;15(1):e0227519. doi: 10.1371/journal.pone.0227519 (PMC6948754; doi:10.1371/journal.pone.0227519)
Supplement: S2 File — (DOCX) [file pone.0227519.s002.docx]

| **A. Demographic Profile**  This section is about yourself and your teaching history. | | |
| --- | --- | --- |
| A1 | Interviewee Code |  |
| A2 | Date of Interview |  |
| A3 | School |  |
| A4 | Your position at this school: |  |
| A5 | How long have you been teaching at this school? |  |
| A6 | How long have you been teaching? |  |
| A7 | Your highest level of education: |  |
| A8 | Your age: |  |
| A9 | Your sex: |  |

**INSTRUCTIONS**

- This is a questionnaire to measure the organisational readiness of your school to implement the CIRCLE of Life Program.
- Please note that there are no right or wrong answers. Please be honest. All answers will remain confidential.
- Tick the correct boxes. Please also add in your comments when asked to elaborate.
- If you would like to change an answer, please put a strike through the incorrect answer and re-tick the correct answer.
- This questionnaire should take you about 10 minutes to complete.
- Please answer all questions.

**B. Health Status of School**

The following questions are about the general health of learners at this school.

| **B1** | In your opinion to what extent does the health and lifestyle of a child affect learning in the classroom?   - To a very great extent - To a great extent - To a moderate extent - To a slight extent - Not at all |
| --- | --- |
| **B2** | To what extent are disease-related problems seen at this school?  *(disease-related means HIV, TB, asthma, diabetes, obesity, malnutrition etc.)*   - To a very great extent - To a great extent - To a moderate extent - To a slight extent - Not at all |
| **B3** | Please list some of the disease-related problems (e.g. HIV, obesity, malnutrition, diabetes, etc.) that the learners at this school possibly present with? |
| **B4** | Your response to B3 above, is based on (choose all appropriate):   - Medical sick notes - Parents notifying you - Absenteeism rates in classes - Your own, family and teaching experiences - Performance in classes, tests and exams - Other (please state): ________________________________________ |
| **B5** | To what extent is high-risk behaviour (e.g. smoking, alcohol, drugs, etc.) a problem among learners at this school?   - To a very great extent - To a great extent - To a moderate extent - To a slight extent - Not at all |
| **B6** | To what extent is obesity a problem among learners at this school?   - To a very great extent - To a great extent - To a moderate extent - To a slight extent - Not at all |
| **B7** | In your opinion, to what extent are learners physically active at this school?   - To a very great extent - To a great extent - To a moderate extent - To a slight extent - Not at all |

**C. Focus of the Program**

These questions are about the CIRCLE of Life Initiative.

| **C1** | In your opinion to what extent are health programs such as the CIRCLE of Life needed at schools?   - To a very great extent - To a great extent - To a moderate extent - To a slight extent - Not at all (State why)________________________________________ |
| --- | --- |
| **C2** | To what extent was there **communication** with staff regarding the CIRCLE of Life Initiative?   - To a very great extent - To a great extent - To a moderate extent - To a slight extent - Not at all |
| **C3** | To what extent was there **consultation** with staff about the CIRCLE of Life Initiative?   - To a very great extent - To a great extent - To a moderate extent - To a slight extent - Not at all |
| **C4** | In your opinion to what extent do all staff understand the objectives and goals of the program?   - To a very great extent - To a great extent - To a moderate extent - To a slight extent - Not at all (State why)________________________________________ |
| **C5** | In your opinion to what extent are these objectives and goals achievable?   - To a very great extent - To a great extent - To a moderate extent - To a slight extent - Not at all (State why)________________________________________ |
| **C6** | In your opinion to what extent will the CIRCLE of Life Initiative influence behaviour change in **learners**?   - To a very great extent - To a great extent - To a moderate extent - To a slight extent - Not at all (State why)________________________________________ |
| **C7** | In your opinion to what extent will the CIRCLE of Life Initiative influence behaviour change in **parents**?   - To a very great extent - To a great extent - To a moderate extent - To a slight extent - Not at all (State why)________________________________________ |
| **C8** | In your opinion to what extent will other staff members at this school think that the CIRCLE of Life Initiative is important?   - To a very great extent - To a great extent - To a moderate extent - To a slight extent - Not at all (State why)________________________________________ |
| **C9** | In your opinion to what extent will this school implement the CIRCLE of Life Initiative effectively?   - To a very great extent - To a great extent - To a moderate extent - To a slight extent - Not at all (State why)________________________________________ |
| **C10** | In your opinion to what extent will the CIRCLE of Life Initiative be a top priority at this school?   - To a very great extent - To a great extent - To a moderate extent - To a slight extent - Not at all (State why)________________________________________ |

**D. Educational Support for Implementation of the Program**

These questions are about training for the CIRCLE of Life Initiative.

| **D1** | How confident are you that teachers will receive the relevant training and support to enable them to fulfil the activities of the CIRCLE of Life Initiative?   - Extremely confident - Very confident - Moderately confident - Slightly confident - Not at all |
| --- | --- |
| **D2** | How confident are you that teachers will receive the relevant training material and notes to enable them to fulfil any of the activities with the CIRCLE of Life Initiative?   - Extremely confident - Very confident - Moderately confident - Slightly confident - Not at all |

**E. Recognition for Implementation of Program**

These questions are about recognition for implementing the CIRCLE of Life Initiative.

| **E1** | In your opinion to what extent will the staff that implement the program be seen as health specialists?   - To a very great extent - To a great extent - To a moderate extent - To a slight extent - Not at all |
| --- | --- |
| **E2** | In your opinion to what extent will the staff that implement the program be held in high esteem in this school?   - To a very great extent - To a great extent - To a moderate extent - To a slight extent - Not at all |
| **E3** | In your opinion to what extent will the staff that implement the program be more likely promoted in rank?   - To a very great extent - To a great extent - To a moderate extent - To a slight extent - Not at all |

**F. Rewards for Implementation of the Program**

These questions are about being rewarded for implementing the CIRCLE of Life Initiative.

| **F1** | How would this program impact on you and the school (choose all relevant responses)?   - Increase work load for teachers - Increase monitoring and evaluation for teachers - Increase parent interaction - Reduce absenteeism at school - Benefit the learners - Benefit teachers - Benefit parents - Benefit the community - No impact at all - Other: (State)_____________________________________________ |
| --- | --- |

**G. Openness of Teaching Staff**

These questions are about the openness of teaching staff.

| **G1** | In your opinion to what extent are staff at this school adaptable to change?   - To a very great extent - To a great extent - To a moderate extent - To a slight extent - Not at all (State why)________________________________________ |
| --- | --- |
| **G2** | In your opinion to what extent are staff at this school flexible to change?   - To a very great extent - To a great extent - To a moderate extent - To a slight extent - Not at all (State why)________________________________________ |
| **G3** | In your opinion to what extent are staff at this school open to new ideas?   - To a very great extent - To a great extent - To a moderate extent - To a slight extent - Not at all (State why)________________________________________ |
| **G4** | In your opinion to what extent are staff at this school concerned about the health of learners?   - To a very great extent - To a great extent - To a moderate extent - To a slight extent - Not at all |
| **G5** | In your opinion to what extent are staff at this school concerned about the health of the community?   - To a very great extent - To a great extent - To a moderate extent - To a slight extent - Not at all |

**H. Preparation for Change**

These questions are about the openness of teaching staff to change. Please read the following statements and indicate whether you agree, somewhat agree, neither agree nor disagree, somewhat disagree or disagree with them.

| **H1** | The CIRCLE of Life Initiative program is well planned for the school environment.   - Agree - Somewhat Agree - Neither Agree nor Disagree - Somewhat Disagree - Disagree (State why)________________________________________ |
| --- | --- |
| **H2** | The staff will want to implement such a change.   - Agree - Somewhat Agree - Neither Agree nor Disagree - Somewhat Disagree - Disagree (State why)________________________________________ |
| **H3** | The staff will do whatever it takes to implement this program.   - Agree - Somewhat Agree - Neither Agree nor Disagree - Somewhat Disagree - Disagree (State why)________________________________________ |
| **H4** | The staff will be determined to implement this program.   - Agree - Somewhat Agree - Neither Agree nor Disagree - Somewhat Disagree - Disagree (State why)________________________________________ |
| **H5** | The staff in this school will be motivated to implement this program.   - Agree - Somewhat Agree - Neither Agree nor Disagree - Somewhat Disagree - Disagree (State why)________________________________________ |
| **H6** | The teachers who work here will be committed to implementing the program.   - Agree - Somewhat Agree - Neither Agree nor Disagree - Somewhat Disagree - Disagree (State why)________________________________________ |
| **H7** | The staff will coordinate tasks so that implementation goes smoothly.   - Agree - Somewhat Agree - Neither Agree nor Disagree - Somewhat Disagree - Disagree (State why)________________________________________ |
| **H8** | The staff will keep track of progress when implementing this program.   - Agree - Somewhat Agree - Neither Agree nor Disagree - Somewhat Disagree - Disagree (State why)________________________________________ |
| **H9** | The staff can handle the challenges that might arise from implementing this change.   - Agree - Somewhat Agree - Neither Agree nor Disagree - Somewhat Disagree - Disagree (State why)________________________________________ |
| **H10** | The teachers who work here feel confident that the school can get the learners invested in implementing this program.   - Agree - Somewhat Agree - Neither Agree nor Disagree - Somewhat Disagree - Disagree (State why)________________________________________ |
| **H11** | The staff can support learners and parents as they adjust to the change in this program.   - Agree - Somewhat Agree - Neither Agree nor Disagree - Somewhat Disagree - Disagree (State why)________________________________________ |
| **H12** | The staff can manage the politics of implementing this change.   - Agree - Somewhat Agree - Neither Agree nor Disagree - Somewhat Disagree - Disagree (State why)________________________________________ |

**I. General**

Please give me your opinion on the questions that follow.

| **I1** | How would you recommend that the researcher get the staff involved and motivated to successfully carry out the implementation? Please elaborate. |
| --- | --- |
| **I2** | Do you foresee any problems (such as culture, policy, procedures, resources and structure) in the implementation of this program? Please elaborate. |
| **I3** | What do you think will be critical to the success of the program? Please elaborate. |
| **I4** | What do you think could limit the success of the program? Please elaborate. |
| **I5** | What further consultation or communication would you like to have from the researcher? |
| **I6** | Do you have any other comments, questions or concerns? |

**Thank you.**
